# Supplementary material for: High-yield vesicle-packaged recombinant protein production from E. coli
Source: Cell Rep Methods. 2023 Feb 2;3(2):100396. doi: 10.1016/j.crmeth.2023.100396 (PMC10014274; doi:10.1016/j.crmeth.2023.100396)
Supplement: Document S1. Figures S1–S4 and Table S1 [file mmc1.pdf]

Cell Reports Methods, Volume *3*

Supplemental information

High-yield vesicle-packaged recombinant protein production from *E. coli*

Tara A. Eastwood, Karen Baker, Bree R. Streather, Nyasha Allen, Lin Wang, Stanley W. Botchway, Ian R. Brown, Jennifer R. Hiscock, Christopher Lennon, and Daniel P. Mulvihill

Supplemental Items

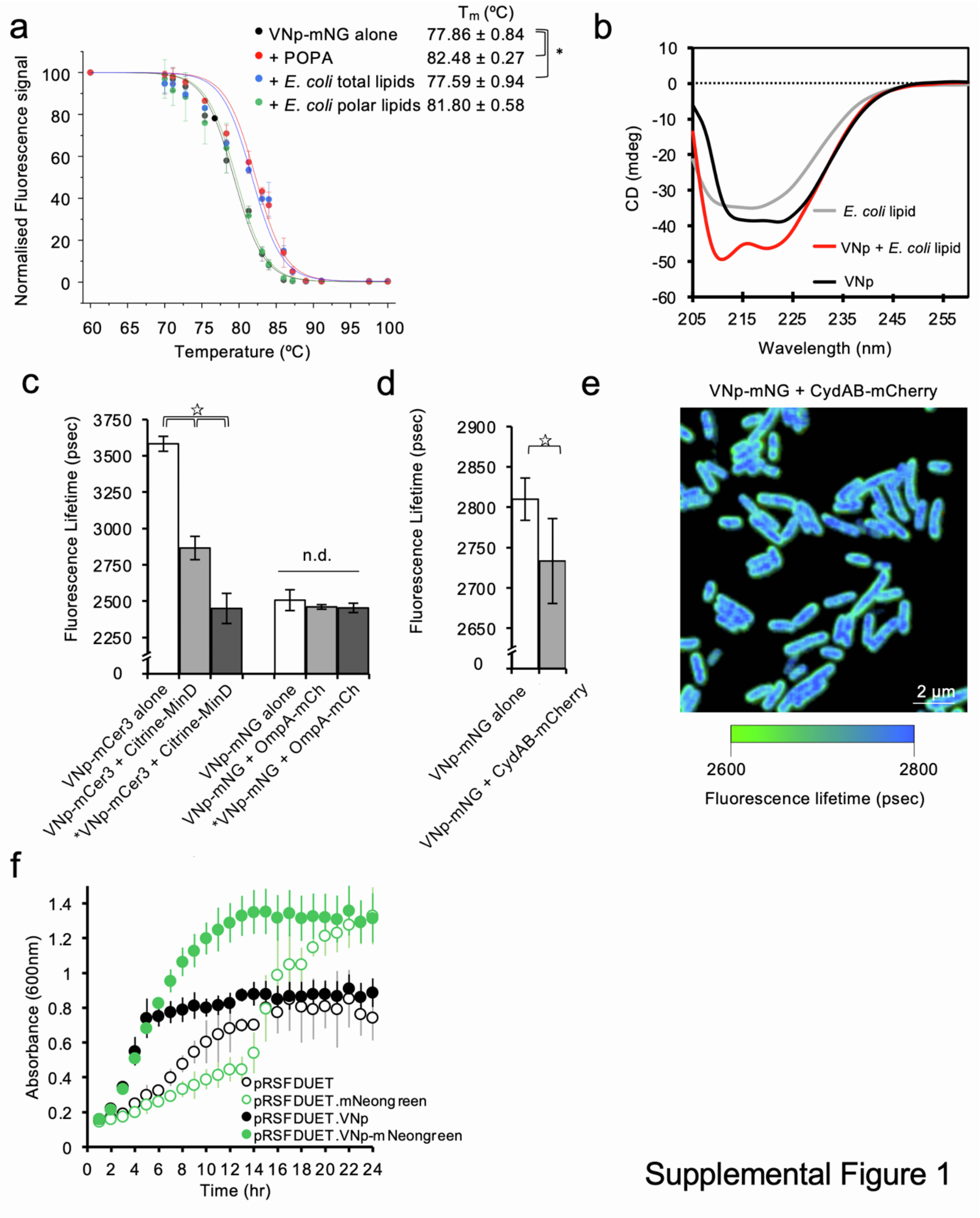

Supplemental Figure 1

SUPPLEMENTAL FIGURE 1. VNp interacts with the inner *E. coli* membrane. Related to Figure 1 and STAR Methods. Thermal shift (a) and Circular Dichroism (b) assays were used to confirm interaction between the VNp and membrane composed of *E. coli* membrane lipids *in vitro.* (a) Interaction with the membrane increases thermal stability of a membrane associated fluorophore. The thermal shift assay was used to examine the impact of different lipid membranes upon fluorophore signal from a fluorescent protein (mNeongreen) when fused to the VNp, a potential membrane binding protein. Average Thermal shift mNeongreen fluorescence curves were calculated for VNp-mNeongreen alone (black), and VNp-mNeongreen in the presence of 100 nm vesicles composed of phosphatidic acid (red), or mixtures of either total (blue) or polar (green) *E.coli* lipids. The shift to the right signifies the VNp-mNeongreen interacts with membranes composed of phosphatidic acid or a mixture of total *E. coli* lipids. (b) Circular Dichroism was used to examine the impact *E. coli* membrane binding has upon the predicted alpha-helical VNp structure. The graph shows averaged CD spectra of VNp alone (black), total *E. coli* lipid vesicles alone (grey), or from a mixture of VNp and *E. coli* lipid vesicles (red). The relative broad negative CD spectra peaks at 208 nm and 222 nm, observed in the mixture of VNp and *E. coli* lipid membrane are consistent with single α-helical structures, and these spectra show that the VNp alpha-helix is stabilised upon interaction with *E. coli* membrane lipid vesicles. (c) Single-photon and (d-e) Multi-photon Fluorescence Lifetime Imaging based Fluorescence Resonance Energy Transfer Microscopy (FLIM-FRET) was used to examine physical interactions between VNp and the *E. coli* inner and outer membranes *in vivo*. Cerulean3 (VNp-Cer3) or mNeongreen (VNp-mNG) fluoreophores were used as donors, and Citrine (Citrine-MinD) or mCherry (OmpA-mCherry/CydAB-mCherry) were used as acceptors. FRET dependent reduction in the fluorescence lifetime of the donor indicates physical interaction (< 10 nm) between proteins (☆ - 99.99% confidence levels). (c) Histogram of donor fluorophore Fluorescence lifetimes of *E. coli* cells expressing VNp donor fluorophore fusions (VNp-Cer3 / VNp-mNG) either alone or with acceptor fluorophore labelled inner (Citrine-MinD) or outer (OmpA-mCherry) membrane proteins indicate VNp interacts with the *E. coli* inner membrane. (d) The histogram of mNeongreen fluorescence lifetime within *E. coli* cells expressing an mNeongreen donor fluorophore VNp fusion (VNp-mNG alone or in combination with the mCherry labelled CydAB inner membrane complex confirmed interaction between VNp and the inner membrane. (e) mNeongreen fluorescence Lifetime micrograph of VNp-mNG CydAB-mCherry expressing cells (from d) illustrates the reduced fluorescence lifetime of the VNp-mNeongreen at the cell membrane, where CydAB is located. The reduction in lifetime length reflected in change from blue (2.8 ns) to green (2.6 ns). (f) To assess the impact of VNp expression on *E. coli* viability averaged growth curves were generated from 4 independent replicate cultures of BL21(DE3) *E. coli* cells containing either an empty pRSFDUET vector (empty black circles), pRSFDUET.mNeongreen (empty green circles), pRSFDUET.VNp (filled black circles) or pRSFDUET.VNp.mNeongreen (filled green circles). Cells were grown at 37 ºC in TB supplemented with kanamycin and 20 µg/ml IPTG on the same 96-well plate. Vesicle containing centrifuged media had no measurable difference in absorbance at 600 nm, indicating the observed changes in absorbance are due to increase in cell number. These data illustrate expression of VNp or a VNp fusion does not negatively impact bacterial growth over a 24 hour period.

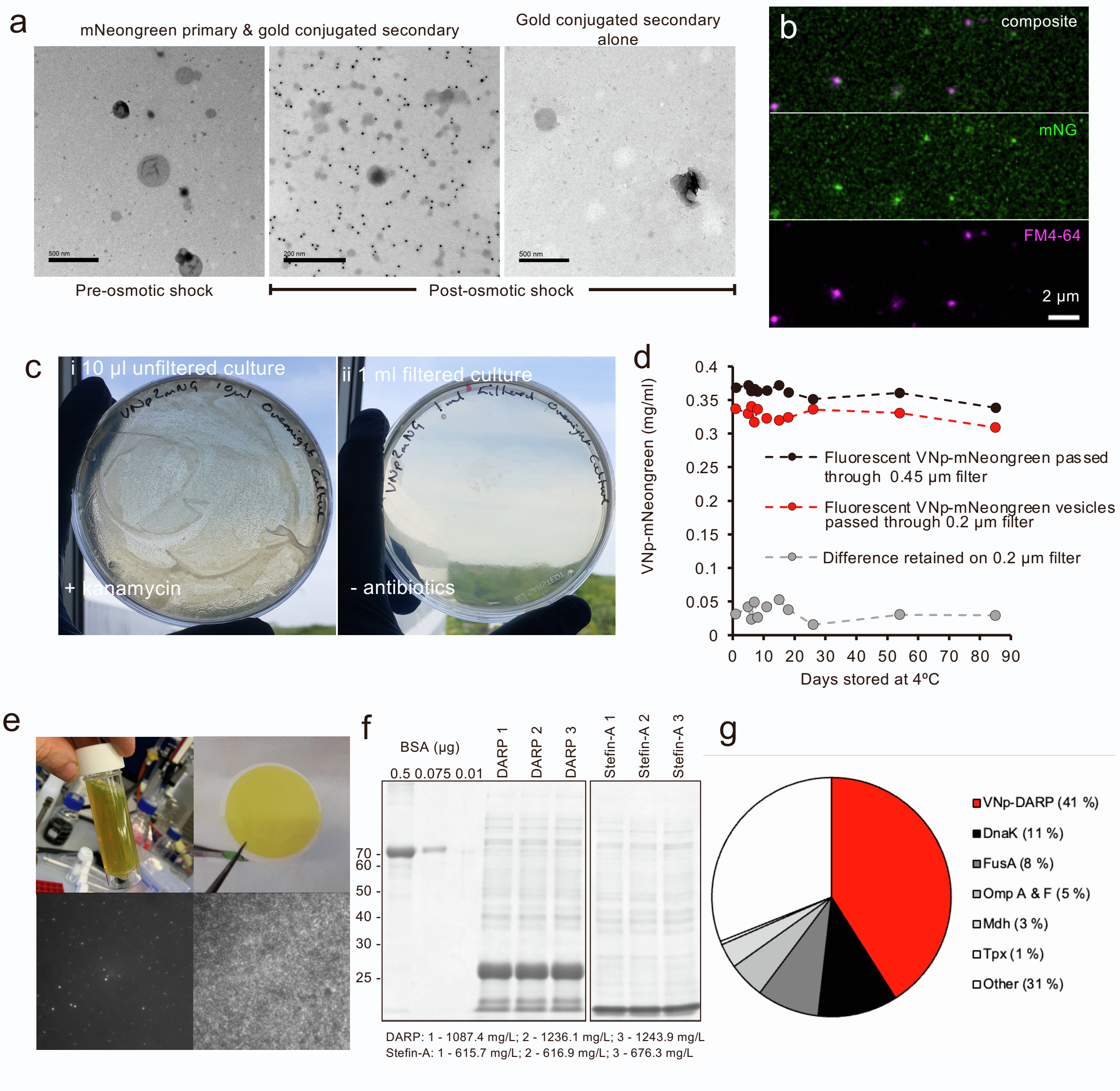

Supplemental Figure 2

Supplemental Figure 2: VNp fusion protein is contained within the lumen of isolated recombinant vesicles. Related to STAR Methods. VNp-mNeongreen containing vesicles were filter purified from media of an overnight culture of BL21 DE3 pRSFDUET1-VNp-mNeongreen cells and mounted onto EM grids and subjected to anti-mNeongreen immuno EM analysis. VNp-mNeongreen dependent gold labelled densities bound to mNeongreen released from vesicles upon osmotic shock from resuspension in water. The lack of densities in control samples subjected to either immuno-analysis prior to bursting, or burst vesicles processed in the same way but without primary anti-mNeongreen antibodies illustrate the mNeongreen is located exclusively within the lumen of the vesicles. (b) Wide field image of VNp-mNeongreen (green) vesicles subjected to FM4-64 (magenta) membrane staining. (c) Test illustrating exclusion of viable *E. coli* cells from the vesicle containing filtrate. 10 µl of total culture (i) and 1 ml of 0.45µm media filtrate (ii) from an overnight culture of VNp-mNG expressing *E. coli* cells were plated out onto LB (ii) or LB supplemented with kanamycin (i) and incubated overnight at 37 ºC. (d) The VNp induced vesicle provided a stable environment for storage of VNp-fusions. VNp-mNeongreen containing vesicles, filter purified from media of an overnight culture of BL21 DE3 pRSFDUET1-VNp-mNeongreen cells were stored at 4 ºC. Overall mNeongreen fluoresecence and the fraction of mNeongreen fluorescence within vesicles retained by a 0.2µm filter did not vary over time, indicating stability of vesicles and folded mNeongreen protein within them. (e) Universal containing centrifuged media from an overnight culture of VNp-mNeongreen expressing cells (upper left) concentrated upon 0.1 µm filter (top right), and associated wide-field flourescence images of mNeongreen containing vesicles. (f) SDS-PAGE gel of BSA quantitation controls and 8 µl of centrifuged media fractions from VNp-fusion induction cultures from independent biological repeats. These samples were subject to tryptic digest and proteomic analysis (example VNp-DARP1 sample1 shown in Table S1). (g) Pie chart showing typical composition of VNp-fusion containing vesicles as determined from the proteomic analysis .

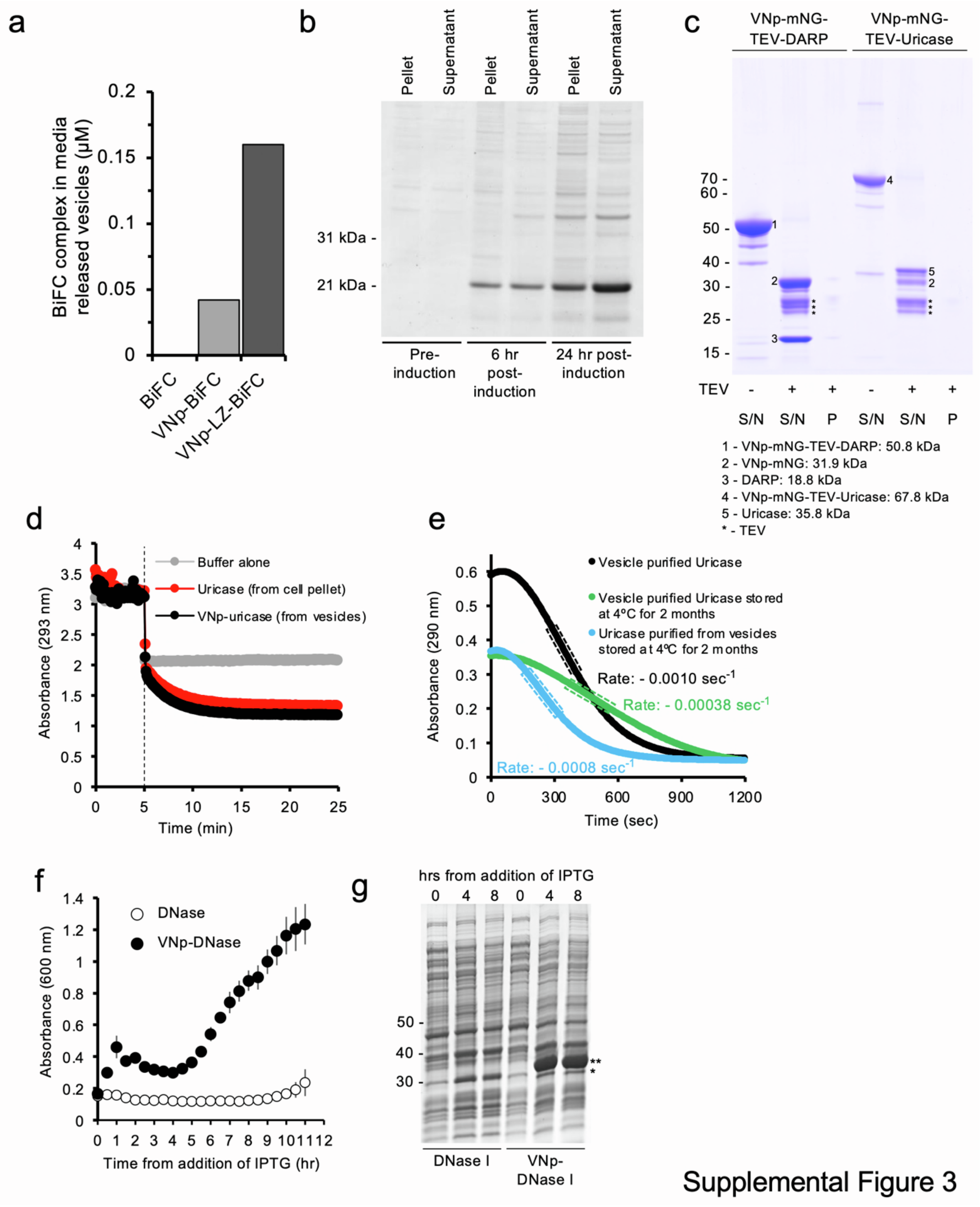

Supplemental Figure 3

Supplemental Figure 3: Functional VNp-fusions are targeted to the VNp-vesicles. Related to STAR Methods. (a) Bimolecular Fluorescence Complementation dependent Fluorescence between the amino (VenusN154) and carboxyl (VenusC155) was used to establish heterocomplexes between different VNp fusions could be targeted to and isolated from the same vesicle. VenusN154-VenusC155 BiFC dependent fluorescence (515 nm) from vesicles isolated from BL21(DE3) *E. coli* expressing either VenusN154 & VenusC155 (white); VNp-VenusN154 & VNp-VenusC155 (grey); or VNp-LZ-VenusN154_VNp-LZ-VenusC155 (black) was used to establish the concentration of the BiFC complex within isolated vesicles. (b) Coomassie stained SDS-PAGE gel of cell pellet and filtered media supernatant samples taken from a 24 hr 15 L fermenter culture of BL21-DE3 cells expressing VNp-DARP. Obtained VNp-DARP yields at end of fermentation were 1.4 g/L in media and 3.1 g/L in cell pellets. (c) Purified VNp-mNG-TEV-DARP and VNp-mNG-TEV-Uricase were digested with TEV protease. The resultant cleaved proteins were not detectable within the pellet fraction (P), and remained in the supernatant (S/N) fraction after centrifugation at 13,200 RCF. (d) The enzymatic activity of Uricase either isolated from a cell pellet using conventional methods (red) or from VNp-uricase induced vesicles (black) was examined to compare functionality of each protein. A buffer only control (grey) shows dilution dependent change in baseline 293 nm absorbance of uric acid. Uricase enzyme / buffer was added to the uric acid substrate after a 5 min equilibration (dashed line). The activity of uricase isolated from cell pellet or VNp-uricase containing vesicles were equivalent. (e) To examine the stability of uricase enzyme activity from protein stored within VNp-uricase vesicles, uricase activity of either fresh vesicle purified VNp-uricase (black; the same vesicle purified VNp-uricase stored in reaction buffer at 4 ºC for 2 months (green); or freshly purified from vesicles that had been stored at 4 ºC for 2 months (blue), were measured using stopped-flow. Rates were determined from steady state regions (highlighted by boxes) of averaged curves and show while uricase stored in buffer exhibited 38% of the original activity, uricase stored within vesicles retained 80% of the original enzymatic activity. (e & f) To establish whether vesicular compartmentalisation of VNp fusions allowed expression of toxic proteins the expression and impact on *E. coli* growth of DNAse1 and VNp-DNaseI were compared. (a) Average growth curves and (b) expression profiles of *E. coli* expressing DNase and VNp-DNase show that while DNaseI only had minimal expression, it inhibited growth of the *E. coli* cells. In contrast the VNp-DNAseI expressed cells grew normally and expressed meaningful levels of the fusion protein.Predicted sizes of DNase and VNp-DNase are 30.2 and 34.4 kDa respectively.

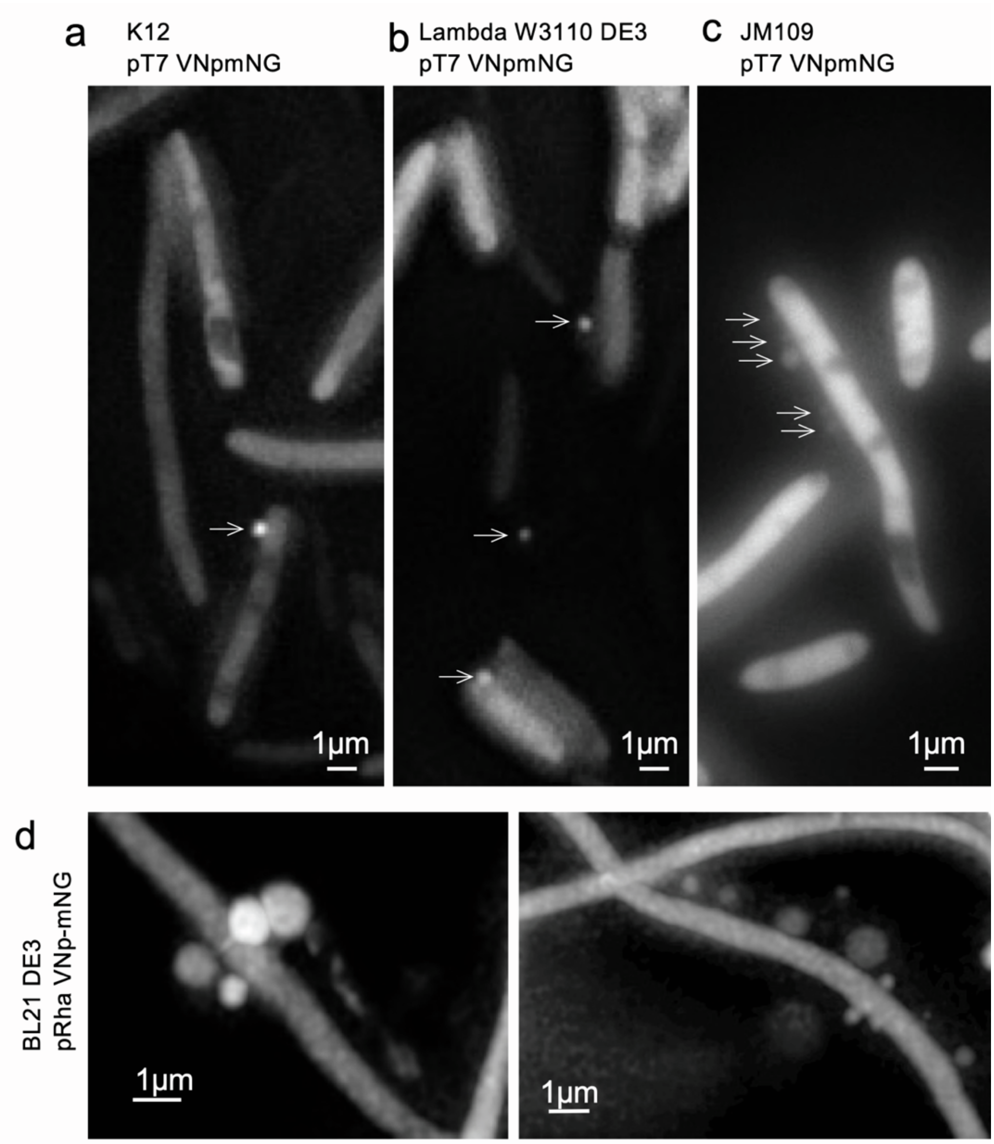

e *Yields of exported protein from W3110 lambda DE3 E. coli cells*

VNp-fusion	Yields (mg protein / L culture)
VNp-DARP	262.8 ± 35.1
VNp-mNeongreen	128.2
VNp6-mNeongreen	478.8
VNp15-mNeongreen	445.4

SUPPLEMENTAL FIGURE 4: VNp induced recombinant protein packaged vesicles using different *E. coli* strains and promoters. Relates to Table 1. Widefield fluorescence images of (a) K12, (b) Lambda DE3 W3110, (c) JM109, and (d) BL21 DE3 *E. coli* strains expressing VNp-mNeongreen from the T7 (a, b, c)) or Rhamnose (d) promoters. (e) Exported Yields of VNp-fusions from W3110 lambda cells.

SUPPLEMENTAL TABLE 1: Proteomic analysis of purified VNp-DARP induced vesicles. Proteomic data from analysis of purified VNp-DARP containing recombinant vesicles (Sample DARP1 from S5b). Vesicle sample was subjected to tryptic digest and mass-spectroscopic analysis. Related to STAR Methods and supplemental figure 2g.

Gene name/Protein	Unique Peptide count	Confidence score	Mass	Raw abundance	Relative abundance
VNp2-DARP	11	94.9	22503	2578835	41.00
dnaK / Chaperone protein DnaK	29	201.2	69172	679166	10.80
fusA / Elongation factor G	38	250.2	77753	530543	8.43
ompF & ompA / Outer membrane porin	12	80.8	39333	309297	4.92
mdh / Malate dehydrogenase	17	121.0	32508	209716	3.33
malE / Maltose/maltodextrin-binding periplasmic protein	10	67.5	43388	166822	2.65
tsf / Elongation factor Ts	13	88.1	30537	115992	1.84
gapA / Glyceraldehyde-3-phosphate dehydrogenase A	7	46.0	35704	109823	1.75
ahpC / Alkyl hydroperoxide reductase C	5	38.3	20876	98732	1.57
grpE / Protein GrpE	2	13.2	21798	65577	1.04
groES / Co-chaperonin GroES	2	12.7	10387	63804	1.01
fbaA / Fructose-bisphosphate aldolase class 2	4	24.4	39375	60187	0.96
eno / Enolase	11	66.9	45712	57412	0.91
slyD / FKBP-type peptidyl-prolyl cis-trans isomerase SlyD	3	20.7	21195	53276	0.85
pgk / Phosphoglycerate kinase	9	58.4	41289	52081	0.83
adk / Adenylate kinase	8	49.6	23643	51143	0.81
tufA / Elongation factor Tu 1	8	53.3	43455	49205	0.78
cysK / Cysteine synthase A	7	58.7	34547	40317	0.64
yncE / Uncharacterized protein YncE	2	10.3	38613	39498	0.63
glnH / Glutamine-binding periplasmic protein	2	11.3	27190	39391	0.63
fabI / Enoyl-[acyl-carrier-protein] reductase [NADH] FabI	5	26.6	28092	39285	0.62
rplL / 50S ribosomal protein L7/L12	1	5.8	12295	38687	0.62
sodB / Superoxide dismutase [Fe]	2	11.2	21323	37129	0.59
frr / Ribosome-recycling factor	1	6.4	20696	31271	0.50
tpx / Thiol peroxidase	3	19.9	18006	30978	0.49
tnaA / Tryptophanase	7	44.0	53173	30304	0.48

lamB / Maltoporin	7	40.1	50026	27768	0.44
cysP / Thiosulfate-binding protein	5	30.2	37615	24683	0.39
glpQ / Glycerophosphodiester phosphodiesterase	4	21.9	40900	24594	0.39
serS / Serine-tRNA ligase	3	17.1	48699	23970	0.38
gpmA / 2_3-bisphosphoglycerate-dependent phosphoglycerate mutase	2	12.6	28556	23620	0.38
glyA / Serine hydroxymethyltransferase	3	16.7	45488	23175	0.37
pal / Peptidoglycan-associated lipoprotein	1	6.3	18881	23114	0.37
aspC / Aspartate aminotransferase	6	35.9	43859	23029	0.37
oppA / Periplasmic oligopeptide-binding protein	8	47.2	61013	22230	0.35
mlaC / Intermembrane phospholipid transport system binding protein MlaC	4	25.2	23963	21725	0.35
crr / PTS system glucose-specific EIIA component	5	29.1	18251	18180	0.29
ahpF / Alkyl hydroperoxide reductase subunit F	2	10.8	56519	17356	0.28
tolB / Tol-Pal system protein TolB	4	25.3	45956	17290	0.27
trxA / Thioredoxin 1	2	11.2	11921	17188	0.27
hisJ / Histidine-binding periplasmic protein	2	12.2	28597	16840	0.27
fkpA / FKBP-type peptidyl-prolyl cis-trans isomerase FkpA	5	28.0	28882	16840	0.27
talB / Transaldolase B	4	24.0	35390	16519	0.26
htpG / Chaperone protein HtpG	5	28.0	71423	16315	0.26
uspA / Universal stress protein A	1	6.7	16123	16070	0.26
gcvP / Glycine dehydrogenase (decarboxylating)	4	21.4	105118	16057	0.26
ridA / 2-iminobutanoate/2-iminopropanoate deaminase	1	6.1	13669	15454	0.25
groEL / Chaperonin GroEL	4	23.0	57500	14988	0.24
potD / Spermidine/putrescine-binding periplasmic protein	2	11.3	38867	14922	0.24
sspA / Stringent starvation protein A	3	17.8	24362	14780	0.23
ackA / Acetate kinase	1	5.4	43633	13679	0.22
dapD / 2_3_4_5-tetrahydropyridine-2_6-dicarboxylate N-succinyltransferase	4	24.0	30063	13347	0.21
glpK / Glycerol kinase	2	11.6	56516	12207	0.19
rbsB / Ribose import binding protein RbsB	2	10.4	30951	11419	0.18

yiaD / Probable lipoprotein YiaD	2	10.5	22254	11288	0.18
pfkB / ATP-dependent 6-phosphofructokinase isozyme 2	1	5.3	32684	11133	0.18
agp / Glucose-1-phosphatase	4	21.3	46025	10709	0.17
asnS / Asparagine-tRNA ligase	5	26.4	52799	9952	0.16
tktA / Transketolase 1	3	17.1	72497	9776	0.16
valS / Valine-tRNA ligase	4	21.3	108649	9747	0.15
lysU / Lysine-tRNA ligase_ heat inducible	4	20.9	57884	9668	0.15
accB / Biotin carboxyl carrier protein of acetyl-CoA carboxylase	1	5.0	16744	8273	0.13
lpp / Major outer membrane lipoprotein Lpp	2	11.7	8381	7809	0.12
yajQ / UPF0234 protein YajQ	1	6.0	18344	7291	0.12
fabA / 3-hydroxydecanoyl-[acyl-carrier-protein] dehydratase	1	5.4	19083	7240	0.12
sucC / Succinate-CoA ligase [ADP-forming] subunit beta	3	16.8	41678	7220	0.11
trxB / Thioredoxin reductase	3	18.4	34851	7083	0.11
hemC / Porphobilinogen deaminase	1	5.8	34080	7060	0.11
degP / Periplasmic serine endoprotease DegP	3	17.0	49468	6886	0.11
yajG / Uncharacterized lipoprotein YajG	1	5.7	21007	6703	0.11
rpsA / 30S ribosomal protein S1	5	27.4	61272	6484	0.10
pykF / Pyruvate kinase I	1	5.3	51072	5739	0.09
moaD / Molybdopterin synthase sulfur carrier subunit	1	5.5	8758	5737	0.09
borD / Prophage lipoprotein Bor homolog	1	5.6	10618	5555	0.09
upp / Uracil phosphoribosyltransferase	1	4.9	22590	5081	0.08
yraP / Uncharacterized protein YraP	1	5.8	20085	5055	0.08
nfuA / Fe/S biogenesis protein NfuA	1	5.5	21226	4614	0.07
pta / Phosphate acetyltransferase	4	20.7	77514	4481	0.07
gloC / Hydroxyacylglutathione hydrolase GloC	1	5.7	24012	4267	0.07
yfbU / UPF0304 protein YfbU	2	10.6	19650	3166	0.05
nusA / Transcription termination/antitermination protein NusA	1	4.9	55042	2767	0.04
tig / Trigger factor	1	5.5	48193	1989	0.03
ppiB / Peptidyl-prolyl cis-trans isomerase B	1	6.2	18268	1654	0.03

Supplemental Video 1: SIM time-lapse of BL21 DE3 containing induced pRSFDuet-1_VNp-LZ_CydAB-mNeongreen. CydAB-mNeongreen labelled inner membranes highlight dynamic movement of VNp-LZ fusion induced membrane bound cytosolic vesicles (100 msec / frame).
