# Supplementary material for: High-yield vesicle-packaged recombinant protein production from E. coli
Source: Cell Rep Methods. 2023 Feb 2;3(2):100396. doi: 10.1016/j.crmeth.2023.100396 (PMC10014274; doi:10.1016/j.crmeth.2023.100396)
Supplement: Document S2. Article plus supplemental information [file mmc3.pdf]

Cell Reports Methods

Article

High-yield vesicle-packaged recombinant protein production from *E. coli*

Graphical abstract

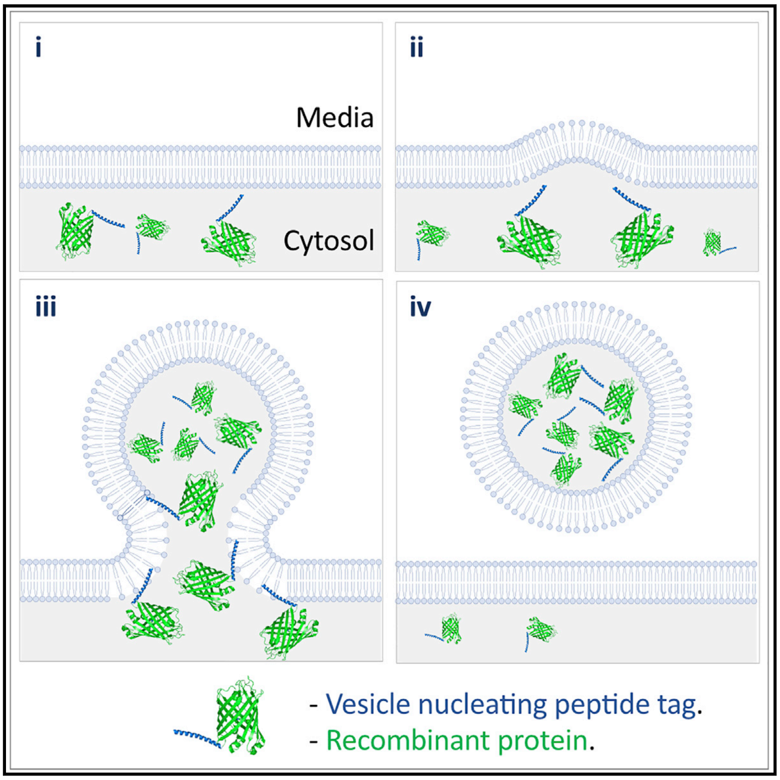

Highlights

- A simple peptide tag generates recombinant-protein-filled vesicles from *E. coli*
- This approach allows production of recombinant protein at high yields
- Enables production of disulfide-bond-containing and toxic proteins
- Recombinant vesicles allow long-term storage of active soluble protein

Authors

Tara A. Eastwood, Karen Baker, Bree R. Streather, ..., Jennifer R. Hiscock, Christopher Lennon, Daniel P. Mulvihill

Correspondence

d.p.mulvihill@kent.ac.uk

In brief

Eastwood et al. describe a system for export of recombinant proteins in membrane-bound vesicles from *E. coli*. A simple peptide tag allows high-yield production of functional proteins within vesicle packages that simplify purification and enable long-term storage. This approach allows production of insoluble, toxic, and otherwise challenging proteins from bacteria.

Eastwood et al., 2023, Cell Reports Methods *3*, 100396
February 27, 2023 © 2023 The Authors.
https://doi.org/10.1016/j.crmeth.2023.100396

CellPress

Cell Reports Methods

CellPress
OPEN ACCESS

Article

High-yield vesicle-packaged recombinant protein production from *E. coli*

Tara A. Eastwood,[1] Karen Baker,[1] Bree R. Streather,[1] Nyasha Allen,[1,2] Lin Wang,[3] Stanley W. Botchway,[3] Ian R. Brown,[1] Jennifer R. Hiscock,[2] Christopher Lennon,[4] and Daniel P. Mulvihill[1,5,*]
[1]School of Biosciences, University of Kent, Canterbury, Kent CT2 7NJ, UK
[2]School of Chemistry and Forensics, University of Kent, Canterbury, Kent CT2 7NJ, UK
[3]Central Laser Facility, Research Complex at Harwell, Science and Technology Facilities Council, Rutherford Appleton Laboratory, Harwell, Didcot, Oxford OX11 0QX, UK
[4]Fujifilm-Diosynth Biotechnologies UK, Ltd., Belasis Avenue, Billingham TS23 1LH, UK
[5]Lead contact
*Correspondence: d.p.mulvihill@kent.ac.uk
https://doi.org/10.1016/j.crmeth.2023.100396

MOTIVATION The ability to reprogram a cell to direct the packaging of specific molecules into discrete membrane envelopes is one of the major challenges in the fields of synthetic biology and recombinant protein today. We thus set out to develop a system to allow the export of vesicle-packaged proteins from *Escherichia coli.* The resultant technology, involving a simple peptide tag, not only simplifies subsequent recombinant protein purification but the controlled packaging into membrane vesicles can be applied to the development of numerous technologies and commercializable products within the biotechnology and medical industries, including generation of recombinant bioreactors, environmental dispersion of biomolecules, and vehicles for drug delivery and vaccination, as well as providing a stable environment for isolation and storage of proteins.

SUMMARY

We describe an innovative system that exports diverse recombinant proteins in membrane-bound vesicles from *E. coli*. These recombinant vesicles compartmentalize proteins within a micro-environment that enables production of otherwise challenging insoluble, toxic, or disulfide-bond containing proteins from bacteria. The release of vesicle-packaged proteins supports isolation from the culture and allows long-term storage of active protein. This technology results in high yields of vesicle-packaged, functional proteins for efficient downstream processing for a wide range of applications from discovery science to applied biotechnology and medicine.

INTRODUCTION

Recombinant protein production has led to a revolution in basic research and biotechnology and biotherapeutic industries and plays a key role in the treatment of a wide range of major diseases. Currently, the majority of commercial recombinant proteins are produced using either bacterial or eukaryotic cell expression systems dependent upon the structural complexity and cell-dependent modifications required to obtain functional protein. The Gram-negative bacteria *Escherichia coli* is an attractive system for recombinant protein production at both academic and industrial scales. It is not only cheap and easy to culture in batches to high densities, but a wide range of strains, reagents, promoters, and tools have been developed to facilitate the production of functional proteins in *E. coli*. In addition, the application of synthetic biology strategies is now overcoming limitations commonly associated with the application of post-translational modifications and folding of complex proteins.[1]

Here, we describe an innovative expression system that induces packaging of a diverse range of recombinant proteins into membrane vesicles in *E. coli*. We identify a simple peptide tag that results in high yields of vesicle-packaged functional proteins and allows compartmentalization of otherwise toxic, insoluble, and disulfide bond-containing proteins, as well as extracellular release of vesicles into the media for efficient downstream processing. These released protein-packed vesicles support rapid isolation from the media and also provide a micro-environment for stable, long-term storage of functional recombinant proteins. Thus, this system provides significant benefit for a wide range of applications from discovery science to applied biotechnology and medicine.

This is an open access article under the CC BY license (http://creativecommons.org/licenses/by/4.0/).

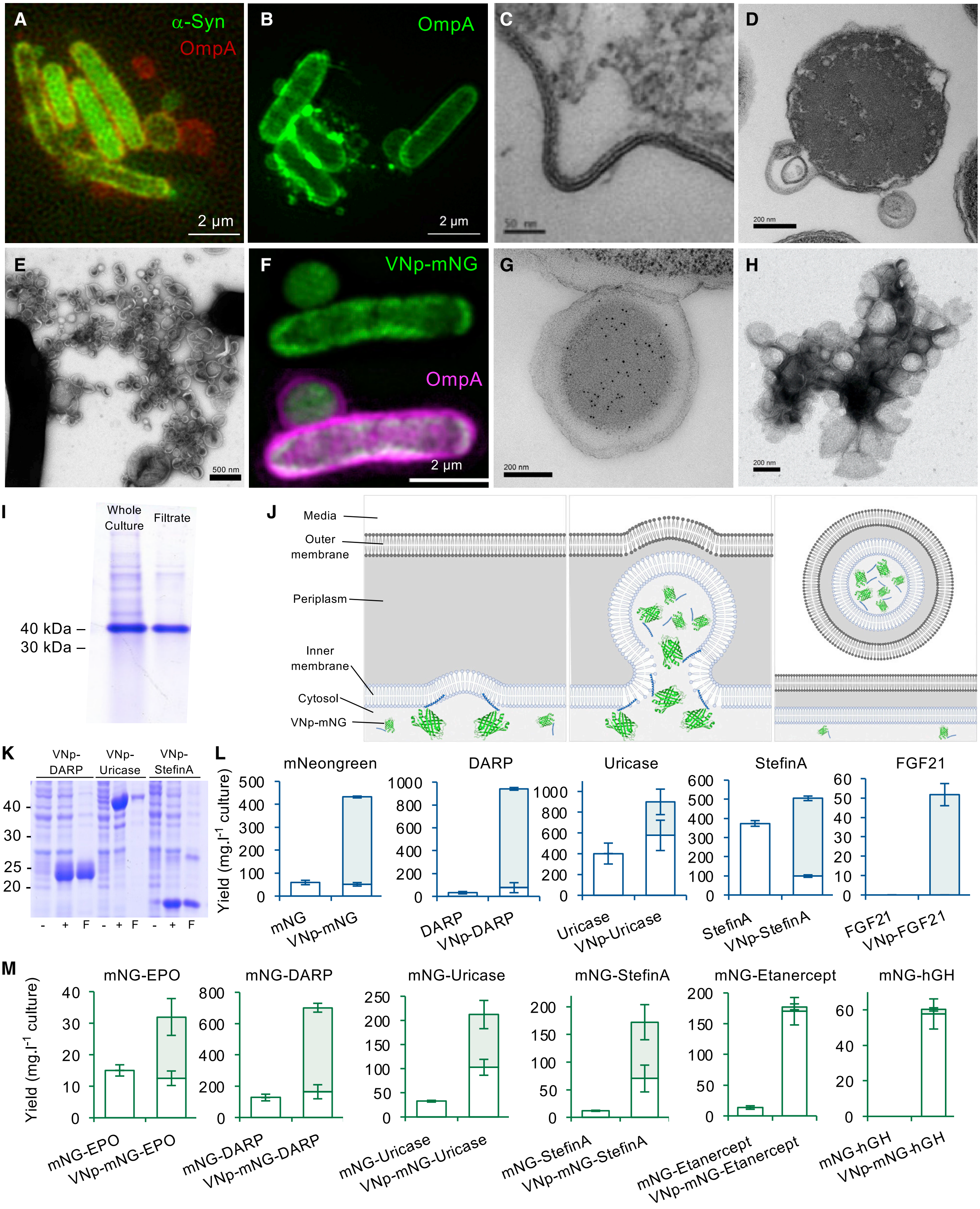

(legend on next page)

RESULTS AND DISCUSSION

During the development of a fluorescence-based drug screen to identify effectors of alpha-synuclein oligomerisation,[2] we serendipitously discovered that recombinant expression of full-length human α-synuclein (αSyn) in *E. coli* brought about the release of extracellular αSyn-containing membrane vesicles, frequently containing the bacterial membrane protein OmpA (Figure 1A). Further analysis revealed that the alpha-helical[3] amino-terminal 38 residues of αSyn are sufficient to bring about the formation and release of OmpA-labeled extracellular membrane-bound vesicles from *E. coli* cells into the culture media (Figure 1B). *In vitro* analysis revealed that this αSyn-derived polypeptide, named here "vesicle nucleating peptide" (VNp), interacts with vesicles composed of reconstituted *E. coli* membrane lipids and subsequently stabilizes its alpha-helical structure[4] (Figure S1). Fluorescence lifetime imaging microscopy (FLIM)-fluorescence resonance energy transfer (FRET) revealed that the VNp fusion specifically associates with the inner *E. coli* membrane *in vivo* (Figure S1), which coincides with the formation and release of recombinant VNp-containing vesicles into the growth media (Figures 1A and 1C–1E). This process occurs without impacting cell growth and so drives large-scale production of vesicles from cells (Figures 1E and S1) to support isolation of recombinant proteins from growth culture media, as well as from cells harvested upon termination of the culture, thus providing significant savings in both time and resource.

Fusion of sequences encoding VNp to those encoding the monomeric fluorescent protein mNeongreen[5] led to the production and export of large VNp-mNeongreen protein vesicles into the culture media (Figures 1F and 1G). Immunoelectron microscopy confirmed the exclusive localization of the mNeongreen cargo within the lumen of the vesicles (Figures 1G and S2). Low-speed centrifugation and subsequent filtration with sterile 0.45 μm polyethersulfone (PES) filters efficiently and effectively isolated the vesicles from bacteria (Figures 1H, 1I, and S2). Average polydispersity indices from dynamic light scattering (DLS) analysis of isolated VNp-induced vesicles were greater than 1, indicating vesicles with a broad distribution of sizes in the culture media. There was no significant difference in the zeta potential (calculated from peak maxima) between day-old (−10.5 mV) and 4-month-old vesicles (−11.1 mV) and no observable significant loss in vesicle-contained VNp-mNeongreen from vesicles over a 3-month period (Figure S2). Thus, the isolated vesicles provide a stable environment for effective long-term protein storage of soluble recombinant protein (Figure S2). The degree of purity of the fusion protein harvested by one-step filtration was determined by mass spectroscopic protein analysis of the isolated vesicles and was found to be sufficient for a very wide range of applications (Figures 1I and S2) while simultaneously supporting subsequent purification after vesicle sonication where necessary. Together, these data support a model of the VNp fusion interacting with the *E. coli* membrane and subsequent incorporation into vesicles that release into the culture media (Figure 1J).

This system provides a simple and attractive mechanism for releasing membrane-packaged recombinant proteins into the media, enabling both enhanced recombinant protein production and subsequent processing. While mNeongreen provided rapid quantification of soluble target protein exported into the media, a wider range of proteins, including a number of model biopharmaceuticals, representing a range of different physical properties and expression challenges (such as membrane binding, disulfide-bond-containing, or otherwise insoluble or toxic proteins; see supplemental information), were used to test the applicability of this technology for the expression of the spectrum of molecules demanded by the life sciences community. Expression of each protein was tested as VNp, or VNp-mNeongreen amino terminal fusions, and compared to the expression of equivalent non-VNp fusion proteins (Figures 1K–1M and S2; Table 1).

The VNp fusion enhanced the expression of each target protein highly effectively and supports the expression of individual proteins ranging from less than 1 kDa (VNp-His6) to 85 kDa (VNp-mNeongreen-etanercept) in size, as well as protein complexes, as demonstrated by fluorescence from pairs of bimolecular fluorescence complementation (BiFC) VNp fusions[6] within exported vesicles (Figure S3). Importantly, VNp fusion enhanced the overall yield of each target protein examined, with yields of almost 1 g soluble protein/liter of shaking flask culture obtained in the case for the designed ankyrin repeat protein DARPin Off7 (DARP) (Table 1). Interestingly, while the addition of the mNeongreen tag was seen to enhance the expression of solubility erythropoietin (EPO), etanercept, and human growth hormone (hGH), the addition of the 25 kDa mNeongreen fluorescent protein tag resulted in a reduction in the overall yield of each model therapeutic protein examined. This is likely to due to a combination of an overall increase in protein size as well as a varying negative effect that mNeongreen can have on the growth of the bacterial cell (Figure S1). In addition, we observed no

Figure 1. Recombinant vesicle formation

(A) SIM fluorescence images of *E. coli* expressing αSyn-mNeongreen (green) and OmpA-mCherry (red) show production of extracellular αSyn-containing membrane vesicles.

(B–D) OmpA-mCherry SIM fluorescence (B) and TEM (C) and (D) images illustrating that VNp induces membrane curvature in *E. coli*.

(E) EM of vesicles generated from VNp-expressing *E. coli* cells that were cultured on prepared grids.

(F) mCherry (magenta) and mNeongreen (green) SIM fluorescence of VNp-mNeongreen OmpA-mCherry-expressing *E. coli* cells.

(G) Anti-mNeongreen immuno-EM of a section through *E. coli* associated VNp-mNeongreen induced vesicle.

(H) TEM images of isolated VNp-mNeongreen-containing vesicles.

(I) Coomassie stained gel of cell culture and filtered media of VNp-mNeongreen-expressing cells.

(J) Schematic of VNp-induced cargo-containing vesicles.

(K) Coomassie stained samples of uninduced and induced cultures or filtered induced cultures of VNp-DARP-, VNp-uricase-, and VNp-stefin A-expressing cells.

(L and M) Average soluble yields per liter of culture derived from cell extracts (empty boxes) or filtered culture media (filled boxes) for each recombinant protein examined. Recombinant proteins lacked (L) or possessed (M) a fluorescent mNeongreen fusion. Errors are SD from ≥3 experimental repeats.

Table 1. Summary of soluble protein yields from shaking flask cultures

Protein	Total yield	Cytosolic	Exported	% export
mNeongreen	59	59 ± 10	ND	0
DARP	32	32 ± 11	ND	0
Uricase	402	402 ± 101	ND	0
Stefin A	374	374 ± 15	ND	0
EPO	0	ND	ND	0
FGF21	0	ND	ND	0
Etanercept	0	ND	ND	0
hGH	0	ND	ND	0
VNp-mNeongreen	472	55 ± 4	417 ± 0	88
VNp-DARP	941	76 ± 44	865 ± 12	92
VNp-uricase	900	577 ± 146	323 ± 123	36
VNp-stefin A	505	99 ± 6	406 ± 123	80
VNp-FGF21	52	ND	52 ± 6	100
VNp-EPO	0	ND	ND	0
VNp-etanercept (10 µg/mL IPTG)	0	ND	ND	0
VNp-hGH (10 µg/mL IPTG)	0	ND	ND	0
mNG-DARP	128	128 ± 21	ND	0
mNG-uricase	33	33 ± 2	ND	0
mNG-stefin A	12	12 ± 1	ND	0
mNG-EPO	15	15 ± 2	ND	0
mNG-FGF21	9	9 ± 1	ND	0
mNG-etanercept (10 µg/mL IPTG)	14	14 ± 3	ND	0
mNG-hGH (10 µg/mL IPTG)	16	16 ± 3	ND	0
VNp-mNG-DARP	701	164 ± 45	537 ± 28	77
VNp-mNG-uricase	212	103 ± 16	110 ± 29	52
VNp-mNG-stefin A	172	70 ± 24	102 ± 32	59
VNp-mNG-EPO	32	13 ± 2	19 ± 6	61
VNp-mNG-anti-GFP_nanobody	194	194 ± 5	0	0
VNp-mNG-FGF21	23	10 ± 1	13 ± 5	57
VNp-mNG-etanercept (10 µg/mL IPTG)	177	170 ± 22	7 ± 5	4
VNp-mNG-hGH (10 µg/mL IPTG)	60	58 ± 8.5	3 ± 1	4
VNp-LZ-mNeongreen	393	56 ± 29	337 ± 17	86
VNp-LZ-hGH	10	ND	10 ± 2	100
VNp-mNeongreen (50 µg/mL IPTG)	411	167 ± 114	241 ± 24	59
VNp-mNeongreen (100 µg/mL IPTG)	287	59 ± 35	227 ± 28	79
VNp (β-isoform)-mNeongreen	390	284 ± 73	106 ± 15	27
VNp (γ-isoform)-mNeongreen	682	252 ± 159	429 ± 155	63
VNp6-mNeongreen	639	53 ± 22	586 ± 15	92
VNp15-mNeongreen	697	76 ± 20	621 ± 38	89
VNp6-DARP	2,194	50 ± 34	2,145 ± 126	97.7
VNp15-DARP	1,632	104 ± 28	1,528 ± 55	93.6
VNp6-stefin A	1,884	43 ± 14	1841 ± 132	97.7
VNp15-stefin A	2,584	320 ± 34	2,264 ± 153	87.6
VNp-mNeongreen (30°C)	490	314 ± 46	176 ± 28	36
VNp6-mNeongreen (30°C)	554	111 ± 25	443 ± 13	80
VNp-mNeongreen (25°C)	358	241 ± 0	117 ± 9	33
VNp6-mNeongreen (25°C)	520	334 ± 10	187 ± 2	36

Yields measured as mg of soluble recombinant protein/liter. Cells grown in shaking flask cultures at 37°C with T7 promoter induced with 20 µg/mL IPTG unless stated otherwise. All cultures had reached stationary phase with an undiluted OD_{600} of ~2 (i.e., equivalent cell densities) at the time of harvesting. Average yields ± SD calculated from ≥3 independent biological repeats. ND, not detectable.

CellPress
OPEN ACCESS

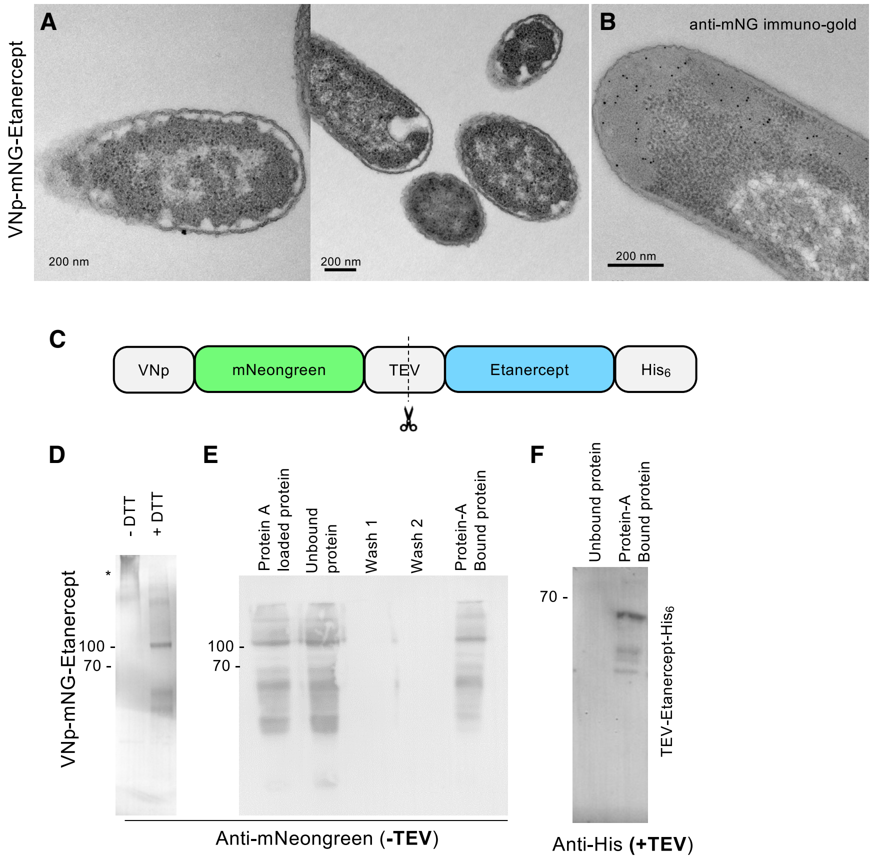

Figure 2. VNp allows expression of functional disulfide-bond-containing IgG1 fusion dimers

(A and B) Conventionally stained (A) and anti-mNeongreen immuno-stained (B) EM serial section images of VNp-mNeongreen-Etanercerpt-induced inward membrane curvature in *E. coli*.

(C) Schematic of the VNp-mNeongreen-TEV-etanercept fusion protein.

(D and E) Anti-mNeongreen western blots illustrating disulfide-bond-dependent ooligomerization (D) and protein A-binding IgG1 functionality (E) of VNp-mNG-etanercept purified from *E. coli*.

(D) VNp-mNG-etanercept disulfide-bond-dependent oligomers (*) are disrupted by the addition of the disulfide-bond-disrupting reducing agent, DTT.

(E) VNp-mNG-etanercept-His_6 fusion was affinity purified from *E. coli* and bound to protein A Dynabeads. Beads were subsequently washed in binding buffer before being boiled in SDS-PAGE loading buffer to release bound proteins. Predicted size of VNp-mNeongreen-etanercept: 83.9 kDa.

(F) Anti-His western blot of wash (unbound) and protein A-bound fractions of TEV cleaved VNp-mNeongreen-TEV-etanercept-His fusion mixed with protein A Dynabeads. Predicted size of etanercept: 52.5 kDa. This illustrates that unlabeled etanercept remains soluble and functional upon removal of the VNp-mNeongreen tag.

significant variation in the size or abundance of the VNp-induced vesicles from cultures expressing different VNp fusions; therefore, the differences in abundances (Table 1) are likely due to differences in expression and packaging efficiency within the vesicle. Tobacco etch virus (TEV) protease cleavage of the VNp-mNeongreen tag from VNp-mNeongreen-TEV-DARP and VNp-mNeongreen-TEV-uricase did not impact the solubility of the resultant purified DARP and uricase proteins (Figure S3), indicating that once expressed, the VNp tag is not necessary for maintaining protein solubility.

The VNp expression system was further validated by illustrating its application to larger-volume fermentation cultures (Figure S3). VNp-DARP expression was induced in *E. coli* over a 24 h period within 15 L fermentation vessels (see STAR Methods for details). Not only was expression and export of the VNp-DARP fusion sustained over the 24 h period (Figure S3), yields of VNp-DARP protein greater than 1.4 g/L were reproducibly obtained, which represented 65% of the total protein observed within the cleared medium fraction.

The versatility of the system was further demonstrated by the production of correctly folded (e.g., mNeongreen) and membrane-binding (FGF21) as well as enzymatically active (uricase) proteins (Figure S3). The vesicle-isolated VNp-uricase was not only as enzymatically active as uricase purified from a cell pellet, but this activity was maintained to a higher degree by VNp-uricase stored within isolated vesicles for 2 months at 4°C when compared with purified protein stored at 4°C in buffer over the same period (Figure S3), highlighting the stable environment the vesicles afford their protein cargo.

The VNp fusion allows production of soluble proteins that are otherwise insoluble or reduce the viability of bacterial cells (e.g., DNase, etanercept, EPO, and hGH) (Table 1; Figure S3). In the case of the disulfide-bond-containing proteins etanercept and hGH,[7,8] the majority of the soluble recombinant protein remained within the cell (Table 1). Electron microscopy (EM) data show that fusing VNp-mNG to etanercept, an anti-inflammatory therapeutic consisting of a fusion between a tumor necrosis factor and immunoglobulin G1 (IgG1), impacts VNp remodeling of the inner membrane to induce VNp-fusion-containing internalized cytosolic membrane structures (Figures 2A and 2B). This tumor necrosis factor (TNF)-IgG1 therapeutic fusion was not only dimeric (disulfide-bond dependent) but also exhibited appropriate ligand-binding properties when isolated from VNp-induced cytosolic vesicles. The ability to bind protein A was maintained upon TEV protease-dependent proteolytic removal of the VNp-mNeongreen fusion (Figures 2C–2E). Similarly, proteolytic cleavage of VNp-mNG from VNp-mNG-TEV-DARP and VNp-mNG-TEV-uricase did not impact the solubility of DARP or uricase (Figure 2F), indicating that VNp is not required to maintain solubility once expressed.

To explore whether dimerization was sufficient to induce internalization of a VNp-fusion protein, stable alpha-helical VNp

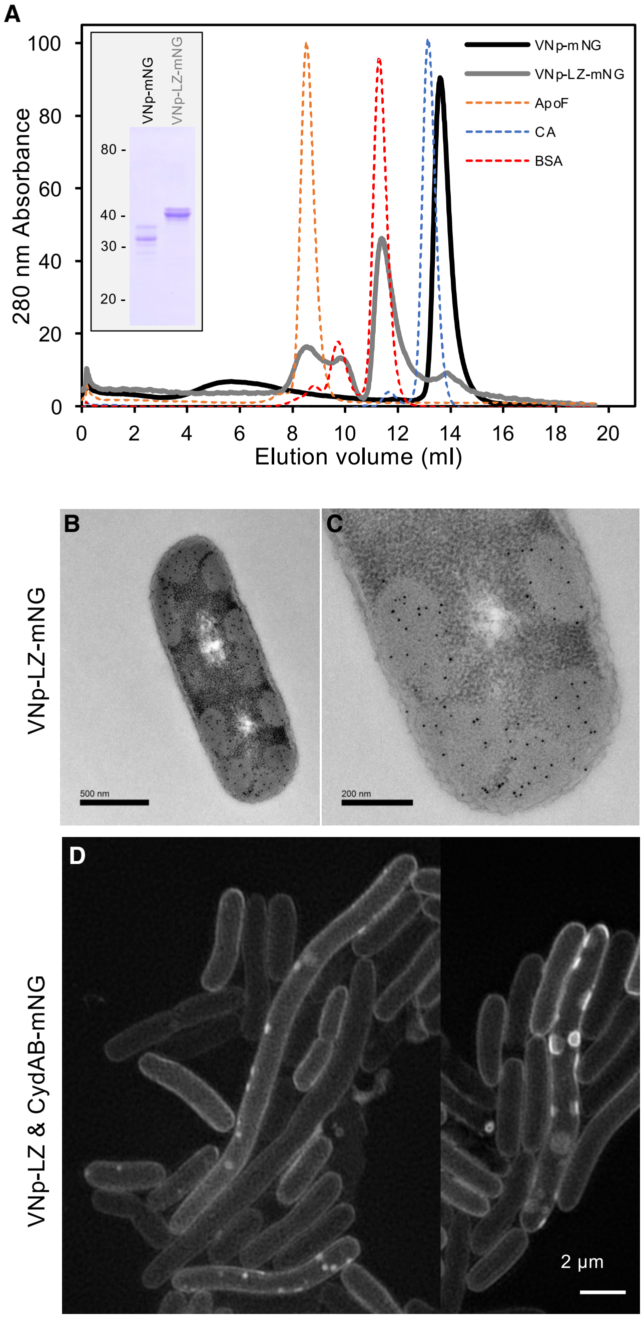

Figure 3. VNp dimers produce VNp-fusion-containing cellular membrane packages

(A) Size-exclusion chromatography profiles of purified recombinant VNp-mNG and VNp-LZ-mNG proteins (inset) confirmed that introduction of a leucine zipper (LZ) motif to the VNp-mNeongreen (mNG) fusion induced stable dimer formation. Each fusion protein as well as protein standards (29 kDa carbonic anhydrase: blue; 66 kDa BSA: red; 443 kDa apoferritin complex: yellow) were run using identical conditions. Whereas the VNp-mNG (black) elution profile was consistent with a monomeric protein, the VNp-LZ-mNG (gray) eluted from the column in earlier fractions consistent with it existing predominantly as a dimer.

(B–D) Anti-mNeongreen immuno-EM images of sections though *E. coli* expressing VNp-LZ-mNeongreen (B) and (C) and SIM images of CydAB-mNeongreen labeled inner membranes in *E. coli* expressing VNp-LZ (D) show that the VNp-LZ dimer concentrates within the lumen of cytosolic inner membrane-bound vesicles.

dimers were created by introducing a leucine zipper (LZ) sequence[9] between VNp and cargo (Figures 3 and S3). Expression of the VNp-LZ fusion induced formation of cytosolic VNp-LZ-fusion-filled vesicular structures, which form from the CydAB[10]-containing inner membrane (Figures 3B–3D; Video S1). While the precise molecular basis is not yet understood, these data illustrate that dimerized VNp fusions promote inward, rather than outward, curvature of the bacterial membrane to provide an attractive method for generating recombinant proteins within cytosolic membrane-bound structures to further facilitate the production of disulfide-bond-containing and otherwise insoluble or toxic proteins from *E. coli*.

Spurred on by the success of this approach, we asked whether simple modifications to the VNp amino acid sequence to modulate the ability to form VNp-fusion-containing vesicles would enhance the exported protein yields. We therefore systematically tested equivalent VNp sequences from the β- and γ-synuclein isoforms (Table 1) as well as a series of constructs generated through modifying charges and side-chain lengths of targeted residues along the helix surface and found that we could not only enhance vesicular export over a wide range of culture temperatures (VNp6) but could also reduce the size of the VNp to 20 residues in length (VNp15) to enhance the export of the target model biopharmaceuticals DARP and stefin A at yields of more than 2.5 g soluble recombinant protein/liter of bacterial flask culture (Table 1). These high yields were reproducible in both academic and industrial environments.

The VNp system exhibits flexibility, as vesicle-packaged proteins can be generated in different *E. coli* strains (e.g., BL21, λ, JM109, and K12 lineages; Figure S4), making it perfectly suited for the production of synthetic proteins with modifications supported by specialist *E. coli* hosts. For example, the VNp system functions in W3110 cells, which allows generation of recombinant-protein-filled vesicles with a reduced immunogenic response.[11] VNp fusions can be expressed from a variety of plasmids (including pUC19- and pBR322-based derivatives), and modulated VNp-fusion expression can be driven from diverse promoters (e.g., T7, rhamnose) and induction levels (Figure S4; Table 1), making this a truly versatile system.

Unlike native outer membrane vesicles that occur naturally in *E. coli*, which form spontaneously in the absence of recombinant protein expression,[12–14] the VNp system described here nucleates vesicle formation through interactions with the inner membrane. In addition, while recombinant proteins are absent from native vesicles released into the media when expressed in *E. coli* cells, the VNp system allows a simple tagging mechanism for targeted recombinant proteins into vesicles (Table 1). This simple peptide fusion increases yields and simplifies downstream processing of a wide range of recombinant proteins from *E. coli*. Importantly, the ease with which otherwise insoluble or toxic proteins can be isolated in milligram or gram quantities suggests that this approach is an attractive starting point for the expression of any recombinant protein of interest. It should be noted that the isolation of protein from VNp-fusion-induced

vesicles is unlikely to provide a route to avoid endotoxin entirely, as proteins are always wrapped in endotoxin during normal secretion processes or homogenizations. Therefore, depending upon the downstream application, the enriched VNp-derived proteins may require further purification. The method has the potential to allow continuous release of protein from extended period cultures in appropriately genetically modified stable expression strains. Another beneficial aspect of this innovation is the stability of proteins and preservation of enzymatic activity when the vesicles are maintained at 4°C. As the use of this system is more broadly adopted and further enhancements and adaptations emerge, its impact can be anticipated to be highly significant. We therefore predict rapid adoption of this versatile system into a wide range of downstream processes and applications.

Limitations of the study

While there is no guarantee this system will enhance production for all proteins, its use resulted in significant increase in yield and solubility for each of the proteins we have tested to date (n > 60). While some of the recombinant-protein-filled vesicles remain cytosolic, it is our experience that these tend to be either dimeric, disulfide-bond-containing, or toxic proteins, which may reflect differences in localized membrane conformation and/or membrane affinity. However, in each of these cases, we also observed enhanced expression and/or functionality of the subsequently expressed proteins. The model depicted in Figure 1J represents a model of how the system works based on current biochemical and imaging data presented in this study. Elucidating the membrane composition of the vesicles and further *in vitro* studies will provide insight into the precise mechanism underlying the formation of the recombinant-protein-filled vesicles described here.

STAR★METHODS

Detailed methods are provided in the online version of this paper and include the following:

- KEY RESOURCES TABLE
- RESOURCE AVAILABILITY
  - Lead contact
  - Materials availability
  - Data and code availability
- EXPERIMENTAL MODEL AND SUBJECT DETAILS
  - *E. coli* strains used in this study
  - Bacterial cell culture and protein induction
- METHOD DETAILS
  - Soluble protein extracts
  - Recombinant vesicle isolation
  - Protein concentration determination
  - Protein isolation from vesicles
  - Circular dichroism (CD)
  - Electrospray LC-MS of proteins
  - In-gel tryptic digest and proteomic analysis of recombinant vesicles
  - Gel filtration assay
  - Lipid binding assay
  - Uricase assay
  - Widefield fluorescence microscopy
  - Fluorescence lifetime imaging microscopy (FLIM)
  - TEM analysis of cells and isolated vesicles
  - TEM thin section analysis of *E. coli* cells
  - Immuno-EM of isolated vesicles
  - Immuno-EM of *E. coli* cells
- QUANTIFICATION AND STATISTICAL ANALYSIS

SUPPLEMENTAL INFORMATION

Supplemental information can be found online at https://doi.org/10.1016/j.crmeth.2023.100396.

ACKNOWLEDGMENTS

The authors thank J. Walklate for assistance undertaking stopped-flow experiments and S. Boxall, M. Geeves, I. Hagan, and D. Manstein for stimulating discussions and comments on the manuscript. This work was supported by the University of Kent and funding from the Biotechnology and Biological Sciences Research Council (BB/S005544/1 and BB/L013703/1_D0101) and Fujifilm-Diosynth Biotechnologies UK Ltd.

AUTHOR CONTRIBUTIONS

T.A.E., K.B., B.R.S., and N.A. performed the experimental studies; L.W. supervised structured illumination microscopy; S.W.B. supervised FLIM; I.R.B. undertook transmission electron microscopy; J.R.H. supervised lipid-binding studies; C.L. undertook fermentation experiments; T.A.E., K.B., C.L., and D.P.M. designed experiments; D.P.M. sought funding and supervised the project; D.P.M. wrote the main drafts of the manuscript, and all authors contributed to editing.

DECLARATION OF INTERESTS

C.L. is an employee at Fujifilm-Diosynth Biotechnologies UK, Ltd. The VNp technology described here is associated with patent application #GB2118435.3.

Received: October 4, 2022
Revised: December 8, 2022
Accepted: January 9, 2023
Published: February 2, 2023

REFERENCES

1. Kim, J.H., Lee, J., Park, J., and Gho, Y.S. (2015). Gram-negative and gram-positive bacterial extracellular vesicles. Semin. Cell Dev. Biol. *40*, 97–104.
2. Eastwood, T.A., Baker, K., Brooker, H.R., Frank, S., and Mulvihill, D.P. (2017). An enhanced recombinant amino-terminal acetylation system and novel in vivo high-throughput screen for molecules affecting α-synuclein oligomerisation. FEBS Lett. *591*, 833–841.
3. Bartels, T., Kim, N.C., Luth, E.S., and Selkoe, D.J. (2014). N-alpha-acetylation of α-synuclein increases its helical folding propensity, GM1 binding specificity and resistance to aggregation. PLoS One *9*, e103727.
4. Fusco, G., Pape, T., Stephens, A.D., Mahou, P., Costa, A.R., Kaminski, C.F., Kaminski Schierle, G.S., Vendruscolo, M., Veglia, G., Dobson, C.M., and De Simone, A. (2016). Structural basis of synaptic vesicle assembly promoted by alpha-synuclein. Nat. Commun. *7*, 12563–12611.
5. Shaner, N.C., Lambert, G.G., Chammas, A., Ni, Y., Cranfill, P.J., Baird, M.A., Sell, B.R., Allen, J.R., Day, R.N., Israelsson, M., et al. (2013). A bright monomeric green fluorescent protein derived from Branchiostoma lanceolatum. Nat. Methods *10*, 407–409.

6. Kodama, Y., and Hu, C.D. (2010). An improved bimolecular fluorescence complementation assay with a high signal-to-noise ratio. Biotechniques *49*, 793–805.
7. Goffe, B., and Cather, J.C. (2003). Etanercept: an overview. J. Am. Acad. Dermatol. *49*, S105–S111.
8. Ultsch, M.H., Somers, W., Kossiakoff, A.A., and de Vos, A.M. (1994). The crystal structure of affinity-matured human growth hormone at 2 Å resolution. J. Mol. Biol. *236*, 286–299.
9. O'Shea, E.K., Klemm, J.D., Kim, P.S., and Alber, T. (1991). X-ray structure of the GCN4 leucine zipper, a two-stranded, parallel coiled coil. Science *254*, 539–544.
10. Safarian, S., Rajendran, C., Müller, H., Preu, J., Langer, J.D., Ovchinnikov, S., Hirose, T., Kusumoto, T., Sakamoto, J., and Michel, H. (2016). Structure of a bd oxidase indicates similar mechanisms for membrane-integrated oxygen reductases. Science *352*, 583–586.
11. Gujrati, V., Prakash, J., Malekzadeh-Najafabadi, J., Stiel, A., Klemm, U., Mettenleiter, G., Aichler, M., Walch, A., and Ntziachristos, V. (2019). Bioengineered bacterial vesicles as biological nano-heaters for optoacoustic imaging. Nat. Commun. *10*, 1114.
12. Blackburn, S.A., Shepherd, M., and Robinson, G.K. (2021). Reciprocal packaging of the main structural proteins of type 1 fimbriae and flagella in the outer membrane vesicles of "wild type" Escherichia coli strains. Front. Microbiol. *12*, 557455.
13. Hong, J., Dauros-Singorenko, P., Whitcombe, A., Payne, L., Blenkiron, C., Phillips, A., and Swift, S. (2019). Analysis of the Escherichia coli extracellular vesicle proteome identifies markers of purity and culture conditions. J. Extracell. Vesicles *8*, 1632099.
14. Thoma, J., Manioglu, S., Kalbermatter, D., Bosshart, P.D., Fotiadis, D., and Müller, D.J. (2018). Protein-enriched outer membrane vesicles as a native platform for outer membrane protein studies. Commun. Biol. *1*, 23.
15. Nji, E., Chatzikyriakidou, Y., Landreh, M., and Drew, D. (2018). An engineered thermal-shift screen reveals specific lipid preferences of eukaryotic and prokaryotic membrane proteins. Nat. Commun. *9*, 4253–4312.
16. Huang, S.H., and Wu, T.K. (2004). Modified colorimetric assay for uricase activity and a screen for mutant Bacillus subtilis uricase genes following StEP mutagenesis. Eur. J. Biochem. *271*, 517–523.
17. Mulvihill, D.P. (2017). Live cell imaging in fission yeast. Cold Spring Harb. Protoc. *2017*. pdb.top090621.
18. Periz, J., Del Rosario, M., McStea, A., Gras, S., Loney, C., Wang, L., Martin-Fernandez, M.L., and Meissner, M. (2019). A highly dynamic F-actin network regulates transport and recycling of micronemes in Toxoplasma gondii vacuoles. Nat. Commun. *10*, 4183–4216.
19. Qiu, H., Gao, Y., Boott, C.E., Gould, O.E.C., Harniman, R.L., Miles, M.J., Webb, S.E.D., Winnik, M.A., and Manners, I. (2016). Uniform patchy and hollow rectangular platelet micelles from crystallizable polymer blends. Science *352*, 697–701.
20. Botchway, S.W., Scherer, K.M., Hook, S., Stubbs, C.D., Weston, E., Bisby, R.H., and Parker, A.W. (2015). A series of flexible design adaptations to the Nikon E-C1 and E-C2 confocal microscope systems for UV, multiphoton and FLIM imaging. J. Microsc. *258*, 68–78.

STAR★METHODS

KEY RESOURCES TABLE

REAGENT or RESOURCE	SOURCE	IDENTIFIER
**Antibodies**		
Rabbit anti-mNeonGreen tag	Cell Signaling Technology	Cat#53061
Mouse anti 6x His	Invitrogen	Cat#15287848
**Bacterial and virus strains**		
BL21 DE3 (DE3)	Lab stock	N/A
DH10b	Lab stock	N/A
W3110	Lab stock	N/A
CLD1040	Lab stock	N/A
JM109	Lab stock	N/A
**Deposited data**		
Raw and analysed data	This study: Kent Data Repository	https://doi.org/10.22024/UniKent/01.01.416.
VNp and LZ peptide sequences		N/A
VNp	MDVFMKGLSKAKEGVVAAAEKTKQGVAEAAGKTKEGVL	N/A
VNp6	MDVFKKGFSIADEGVVGAVEKTDQGVTEAAEKTKEGVM	
VNp15	MDVFKKGFSIADEGVVGAVE	N/A
Uniprot accession numbers of protein cargoes tested in this study		N/A
DARP	Designed Ankyrin Repeat Protein off7 (*Agrobacterium radiobacter*)	B9JMD9
DNase	Deoxyribonuclease I (*Bos taurus*)	P00639
EPO	Erythropoietin (*Homo sapiens*)	P01588
Etanercept	Tumour necrosis factor receptor 1B - IgG1 fusion (*Homo sapiens*)	P20333
FGF21	Fibroblast Growth Factor 21 (*Homo sapiens*)	Q9NSA1
hGH	Somatotrophin (*Homo sapiens*)	P01241
mNeongreen	mNeongreen (*Branchiostoma lanceolatum*)	A0A1S4NYF2
StefinA	Cystatin-A (*Homo sapiens*)	P01040
Uricase	Uricase (*Cyberlindnera jadinii*)	P78609
**Recombinant DNA**		
pRSFDuet-1_VNp-His6	This Study	Addgene 182386
pRSFDuet-1_VNp-mNeongreen	This Study	Addgene 182387
pRSFDuet-1_VNp6-mNeongreen	This Study	Addgene 182388
pRSFDuet-1_VNp15-mNeongreen	This Study	Addgene 182389
pRSFDuet-1_VNp-mNeongreen_OmpA-mCherry	This Study	Addgene 182390
pRSFDuet-1_VNp-mNeongreen_CydAB-mCherry	This Study	Addgene 182391
pETDuet-1_VNp-mCerulean3_Citrine-minD	This Study	Addgene 182420
pRSFDuet-1_VNp-LZ-mNeongreen	This Study	Addgene 182392
pRSFDuet-1_VNp-LZ_CydAB-mNeongreen	This Study	Addgene 182393
pETDuet-1_VenusN154_VenusC155 (BiFC control construct)	This Study	Addgene 87856
pETDuet-1_VNp-VenusN154_VNp-VenusC155 (BiFC construct)	This Study	Addgene 182394
pETDuet-1_VNp-LZ-VenusN154_VNp-LZ-VenusC155 (BiFC construct)	This Study	Addgene 182395
pRSFDuet-1_DARPinOFF7-His_6	This Study	Addgene 182396
pRSFDuet-1_VNp-DARPinOFF7-His_6	This Study	Addgene 182397

(*Continued on next page*)

Continued

REAGENT or RESOURCE	SOURCE	IDENTIFIER
pRSFDuet-1_VNp6-DARPinOFF7-His_6	This Study	Addgene 182398
pRSFDuet-1_VNp15-DARPinOFF7 -His_6	This Study	Addgene 182399
pRSFDuet-1_Uricase-His_6	This Study	Addgene 182400
pRSFDuet-1_VNp-Uricase-His_6	This Study	Addgene 182401
pRSFDuet-1_StefinA -His_6	This Study	Addgene 182402
pRSFDuet-1_VNp-StefinA-His_6	This Study	Addgene 182403
pRSFDuet-1_VNp6-StefinA-His_6	This Study	Addgene 182404
pRSFDuet-1_VNp15-StefinA-His_6	This Study	Addgene 182405
pRSFDuet-1_FGF21-His_6	This Study	Addgene 182406
pRSFDuet-1_VNp-FGF21-His_6	This Study	Addgene 182407
pRSFDuet-1_DNAseI-His_6	This Study	Addgene 182408
pRSFDuet-1_VNp-DNAseI-His_6	This Study	Addgene 182409
pRSFDuet-1_hGH-His_6	This Study	Addgene 182410
pRSFDuet-1_VNp-hGH-His_6	This Study	Addgene 182411
pRSFDuet-1_VNp-LZ-hGH-His_6	This Study	Addgene 182412
pRSFDuet-1_mNeongreen	This Study	Addgene 182413
pRSFDuet-1_mNeongreen-DARPinOFF7-His_6	This Study	Addgene 182414
pRSFDuet-1_VNp-mNeongreen-DARPinOFF7-His_6	This Study	Addgene 182415
pRSFDuet-1_mNeongreen-Uricase-His_6	This Study	Addgene 182416
pRSFDuet-1_VNp-mNeongreen-Uricase-His_6	This Study	Addgene 182417
pRSFDuet-1_mNeongreen-StefinA-His_6	This Study	Addgene 182418
pRSFDuet-1_VNp-mNeongreen-StefinA-His_6	This Study	Addgene 182419
pRSFDuet-1_mNeongreen-Etanercept-His_6	This Study	Addgene 182420
pRSFDuet-1_VNp-mNeongreen-Etanercept	This Study	Addgene 182421
pRSFDuet-1_mNeongreen-Erythropoietin-His_6	This Study	Addgene 182422
pRSFDuet-1_VNp-mNeongreen-Erythropoietin -His_6	This Study	Addgene 182423
Software and algorithms		
ImageJ	National Institutes of Health, Bethesda, Maryland, USA	https://imagej.nih.gov/ij/
Compass Data Analysis software	Bruker	N/A
Origin software	OriginLab	N/A
OmniSEC	Malvern	N/A
Metamorph	Molecular Devices	N/A
Zen software	Zeiss	N/A
SPCImage software v.6.9	Becker and Hickl, GmbH	N/A
Other		
Syringe filter, PES, 0.45 μm	Fisherbrand	Cat#15216869
Millipore Express PLUS 0.45 μm Membrane	Merck	Cat#HPWP04700

RESOURCE AVAILABILITY

Lead contact

Further information and requests for resources and reagents should be directed to and will be fulfilled upon reasonable request by the lead contact Dan Mulvihill (d.p.mulvihill@kent.ac.uk).

Materials availability

Plasmids generated in this study have been deposited to Addgene. Plasmid ID #s 182386–182425.

Data and code availability

- All the raw datasets generated during this study have been deposited at Kent Data Repository and are publicly available as of the date of publication. Microscopy data reported in this study will be shared by the lead contact upon request.

- This paper does not report original code.
- Any additional information required to reanalyze the data reported in this paper is available from the lead contact upon request.

EXPERIMENTAL MODEL AND SUBJECT DETAILS

E. coli strains used in this study

BL21 DE3 F-*ompT hsdSB* (rB–, mB–) *gal dcm* (DE3).
DH10b F-*mcrA* Δ(*mrr-hsd*RMS-*mcr*BC) φ80*lac*ZΔM15 Δ*lac*X74 *rec*A1 *end*A1 *ara*D139 Δ(*ara-leu*)7697 *gal*U *gal*K λ–*rps*L(StrR) *nup*G.
W3110 F- λ - *IN(rrnD-rrnE)1 rph-1*.
CLD1040 F- λ, *IN(rrnD-rrnE)1 rph-1 OmpT*.
JM109 F- *tra*D36 *pro*AB *lac*I^{q}ZΔM15 *end*A1 *rec*A1 *gyr*A96 *thi hsd*R17 (rk–, mk+) *rel*A1 *sup*E44 Δ (*lac-pro*AB).

Bacterial cell culture and protein induction

All bacterial cells were cultured at 37°C using LB (10 g Tryptone; 10 g NaCl; 5 g Yeast Extract (per litre)) and TB (12 g Tryptone; 24 g Yeast Extract; 4 mL 10% glycerol; 17 mM KH_2PO4 72 mM K_2HPO_4 (per litre) media. 5 mL LB starters from fresh bacterial transformations were cultured at 37°C to saturation and used to inoculate 100–500 mL volume TB media, flask cultures that were incubated overnight at 37°C with 200 rpm orbital shaking. Recombinant protein expression from the T7 promoter was induced by addition of IPTG to a final concentration of 20 μg/mL (except etanercept where 10 μg/mL was used) once the culture had reached an OD_{600} of 0.8–1.0). Growth curves were generated from 96 well plate cultures, prepared from late log-phase cultures, diluted into fresh media to an OD_{600} of 0.1 nm at the start of the growth analysis experiment. OD_{600} absorbance values were obtained using a Thermo Scientific Multiscan Go 1510-0318C plate reader and recorded using the SkanIt Software 4.0. at OD_{600} values were taken every 15 minutes for the duration of the experiment, and growth curves generated from averages of 4 individual biological repeats.

METHOD DETAILS

Soluble protein extracts

Cell pellets from 50 mL of culture were resuspended in 5 mL of soluble extract buffer (20 mM TRIS, 500 mM NaCl, pH 8.0), sonicated for a total of 2 min (6 × 20 sec pulses), and cell debris removed by centrifugation at 18,000 rpm (4°C) for 30 min. Target protein concentration was determined using fluorescence of mNeonGreen fusion or gel densitometry. Both techniques were compared directly on the same samples to determine equivalence.

Recombinant vesicle isolation

Vesicles were isolated directly from bacterial cell cultures by passing the culture through a 0.45 μm PES filter. Typical purity and concentration from equivalent volume of culture and filter flow through are shown in Figure 1. Exclusion of viable cells from the vesicle containing filtrate was routinely tested by plating onto LB plates lacking antibiotics and incubating overnight at 37°C (example shown in Figure S2).

Protein concentration determination

Fluorescence scan was used to determine concentration of mNeongreen fusion proteins in vesicle containing media and soluble protein extracts. Absorbance was measured at 506 nm using a Varian Cary 50 Bio UV-Vis spectrophotometer, with measurements from an equivalent empty vector culture used for baseline correction, and concentration determined using an extinction coefficient of 116,000 $M^{-1}cm^{-1}$. Concentration of non-mNeongreen labelled proteins was determined by gel densitometry analysis of triplicate samples run alongside BSA loading standards on Coomassie stained SDS-PAGE gels. Gels were scanned and analysed using Image J software. Concentration was determined by both UV and densitometry for three independent VNp-mNeongreen samples to confirm parity between techniques. Average yields in Figure 1 & Table S1 were calculated (mg target protein/litre culture) from a minimum of 3 independent biological repeats from cultures of BL21 DE3 *E. coli* cells grown in TB media.

Protein isolation from vesicles

Purified VNp induced vesicles were resuspended in ice cold 1xPBS before being sonicated to disrupt vesicle membrane, and release the VNp-fusion protein. In order to further purify carboxyl His_6 tagged recombinant VNp-fusion protein (all recombinant proteins expressed during this study contain carboxyl-terminal His_6 affinity tags), this solution was then mixed in a 1 in 5 dilution of 5 x binding buffer (250 mM TRIS 2.5 M NaCl 5% Triton-X 50 mM Imidazole pH 7.8) before passing over a Ni^{2+}-agarose resin gravity column. Cytosolic recombinant protein was purified by passing soluble protein extracts (supplemented with Imidazole to 20 mM) over the Ni^{2+}-agarose resin gravity column. In both cases matrix bound His-tagged protein was washed, eluted (using imidazole), and dialysed into appropriate storage or assay buffer. Protein identity and amino-terminal acetylation of isolated proteins was confirmed by electrospray mass-spectroscopy.

Circular dichroism (CD)

Measurements were made in 2 mm quartz cuvettes using a Jasco 715 spectropolarimeter. VNp protein and 100 nm extruded vesicles were diluted in CD buffer (10 mM potassium phosphate, 5 mM $MgCl_2$ pH 7.0) to a concentration of 0.4 mg/mL and 0.2 respectively. Broad negative peaks at 208 and 222 nm and a positive peak at < 200 nm are consistent with an α-helical structure.

Electrospray LC-MS of proteins

Electrospray mass spectra were recorded on a Bruker micrOTOF-Q II mass spectrometer. Samples were desalted on-line by reverse-phase HPLC on a Phenomenex Jupiter C4 column (5 μm, 300 Å, 2.0 mm × 50 mm) running on an Agilent 1100 HPLC system at a flow rate of 0.2 mL/min using a short water, acetonitrile, 0.05% trifluoroacetic acid gradient. The eluant was monitored at 214 nm & 280 nm and then directed into the electrospray source, operating in positive ion mode, at 4.5 kV and mass spectra recorded from 500–3,000 m/z. Data was analysed and deconvoluted to give uncharged protein masses with Bruker's Compass Data Analysis software.

In-gel tryptic digest and proteomic analysis of recombinant vesicles

Sample of purified VNp-DARP induced vesicles (shown in Figure S5B) were run on SDS-PAGE, which was subsequently coomassie stained, and the whole sample lane cut out, cut into small pieces, which were subsequently transferred to a 1.5 mL microfuge tube and stored in distilled water at 4°C until processing. The gel particles were washed with 150 μL of freshly made 50 mM NH_4HCO_3: acetonitrile (1:1 ratio) for 15 mins. Liquid was removed and gel fragments resuspended in 150 μL acetonitrile for 15 mins, before liquid was again removed, and gel pieces were resuspended in 100 mL of 10 mM DTT in 50 mM NH_4HCO_3, and incubated for 30 min at 56°C. Gel pieces were centrifuged, and excess liquid removed before incubating for 1 min with 100 μL of acetonitrile, which was again removed and gel fragments were suspended in 100 mL of 55 mM chloroacetamide in 50 mM NH_4HCO_3 and incubated for 20 min at room temp in the dark. Pellets were then centrifuged, the chloroacetamide solution was removed. Gel pieces were subject to subsequent 15 min washes in 150 mL of 50 mM NH_4HCO_3:acetonitrile (1:1), and then150 mL of 50 mM NH_4HCO_3 for 15 min, and liquid was removed by centrifugation after each wash. Gel pieces were then washed for 15 mins with 200 μL of acetonitrile, and then rehydrated in 50 mL of digestion buffer (12.5 mM NH_4HCO_3, 10% acetonitrile) containing 5 ng/mL of trypsin, which was left overnight at room temperature. Upon completion of digestion, 15 mL acetonitrile was added to the sample, where was then sonicate in an ultrasound bath for 15 mins. Gel fragments were isolated by centrifugation and the supernatant collected in a fresh 0.5 mL microfuge tube (A). The gel fragment pellet was resuspended in 30 μL 50% acetonitrile with 5% formic acid, and sonicate in an ultrasound bath for 15 mins, and pellet again isolated by centrifugation and supernatant collected in a fresh 0.5 mL microfuge tube (B). Contents of tube A and B were combined, vacuum dried, and subsequently resuspended in 20 mL of 5% acetonitrile, 0.1% TFA. Samples were run through Pierce C18 Spin Tips and analysis by nano-LCMS.

Gel filtration assay

500 μL of protein samples were loaded to a Superdex 200 Increase 10/300 GL size-exclusion column (GE Healthcare Life Sciences) equilibrated at room temperature in PBS and run at 0.75 mL/min flow rate. Eluted proteins were measured by Viscotek Sec-Mals 9 and Viscotek RI detector VE3580 (Malvern Panalytical).

Lipid binding assay

Affinity of VNp for *E. coli* membrane lipids was established using a thermal shift fluorescence binding assay adapted from.[15] Equivalent assay samples, made up of: 65 μL 3 mg/mL of VNp-mNeongreen, 65 μL 1 mM of 100 nm extruded vesicles composed of the lipid mixture to be tested; 15 μL, 10%OGP; and 5 μL 20 mM Tris-HCl pH 7.0, were prepared in PCR tubes and held at the defined temperature in a gradient PCR machine for 10 minutes. Samples were centrifuged at 18,000 *xg*, and supernatant fluorescence was determined in black 96 well plates (BRAND, Germany) using a BMG Clariostar (BMG Labtech). Fluorescence readings were normalised and used to create a melting curve, where the melting temperature (*Tm*) was determined using Origin software (OriginLab). The final *Tm* value was an average (± s.d) calculated from three independent sample repeats.

Uricase assay

500 μL of 100 mM Tris pH 8.5 with 200 mM Uric acid was placed in a cuvette and OD_{293} measurements were taken over for 4 or 5 minutes. Subsequently either 500 μL of 4.5 mg/mL purified VNp2-Uricase (dialysed into 0.1 M Tris pH 8.5) or dialysis buffer alone was added to the cuvette and OD_{293} measurements taken for 25 mins. (Adapted from[16]).

Widefield fluorescence microscopy

Cells were mounted onto coverslips under <1 mm thick circular LB-agarose(2%) pads, and attached with appropriate spacers onto glass slides, before being visualised on an inverted microscope.[17] All live cell imaging for each sample was completed within 30 mins of mounting cells onto coverslips.

Structured Illumination Microscopy (SIM) was undertaken using a Zeiss Elyra PS 1 microscope with a 100x NA 1.46 oil immersion objective lens (Zeiss α Plan-Apochromat) as described previously.[18,19] Briefly, cells were mounted under thin LB-agarose pads onto high precision No.1.5 coverslips (Zeiss, Jenna, Germany). 488 nm and 561 nm laser were used to illuminate mNeongreen and

mCherry/mScarlet fusions, respectively. The optical filter set consisted of laser blocking filter MBS 405/488/561 as the dichroic mirror, and the dual-band emission filter LBF-488/561. The total of 3 rotations of the illumination pattern were implemented to obtain two-dimensional information. Super-resolution SIM image processing was performed using the Zeiss Zen software. Two colour images were aligned using the same software following a calibration using pre-mounted MultiSpec bead sample.

Fluorescence lifetime imaging microscopy (FLIM)

The one- and two- photon systems used in this work have been previously described.[20] Prior to FLIM data acquisition, protein expression levels were verified using confocal microscope. Here, a Nikon Eclipse C2-Si confocal scan head attached to an inverted Nikon TE2000 or Ti-E microscope was used. mNeongreen and mCherry FP were excited at 491 nm (emission 520/35 nm) and 561 nm (emission 630/50 nm) respectively using an NKT super continuum laser. FLIM images were obtained as follows: 2 photon (950 nm) wavelength light was generated by a mode-locked titanium sapphire laser (Mira F900, Coherent Laser Ltd), producing 180 fs pulses at 76 MHz. This laser was pumped by a solid-state continuous wave 532 nm laser (Verdi 18, Coherent Lasers Ltd). Fluorescence was collected through a BG39 filter for the donor fluorophore. The acceptor was not excited.

For one photon excitation FLIM, the system is equipped with a SuperK EXTREME NKT-SC 470-2000 nm supercontinuum laser (NKT Photonics) which generates at 80 MHz repetition rate with 70 ps pulse width. The desired wavelengths were selected using a SuperK SELECT 29 multi-line tunable filter (NKT photonics). Images were collected through either a 60X 1.2 NA water immersion (Figures S1C and S1D) or 60X 1.49 NA oil immersion (Figure S1E) lens. For both one and two-photon excitation, emission was collected by the same objective through filters (above) and detected with an external hybrid GaAsP (HPM-100-40, Becker & Hickl, Germany), linked to a time correlated single photon counting (TCSPC) module (SPC830, Becker and Hickl, Germany). Photon counts of at least 1,000 used for the multi-exponential analysis. Raw time correlated single photon counting decay curve at each pixel (256 × 256 or higher) of the images were analysed using SPCImage software v.6.9 (Becker and Hickl, GmbH); an incomplete single exponential fit model with a laser repetition time value of 12.5 ns was used for the decay curve fitting. Lifetime values with $\chi 2$ between 0.8 and 1.3 were taken as a good exponential decay fit.

TEM analysis of cells and isolated vesicles

Negative stained TEM samples of cells and vesicles were prepared in one of two ways.10 µL of *E. coli* cells expressing VNp-mNeongreen from an overnight culture was placed onto a formvar/carbon coated 400mesh gold grid and incubated in a humid chamber at 37°C to allow vesicle formation. Recombinant vesicles isolated from a culture of *E. coli* expressing VNp-mNeongreen were placed onto a formvar/carbon coated 600mesh copper grid and left for 5 mins at room temperature to allow vesicles to settle onto the surface. Both samples were then fixed in 2.5% glutaraldehyde in 100 mM sodium cacodylate buffer pH 7.2 (CAB) for 10 minutes. Grids were then washed in 100 mM CAB and milliQ water. Grids were then dried and negative stained for 5 seconds in 2% aqueous uranyl acetate.

TEM thin section analysis of *E. coli* cells

E. coli expressing VNp-mNeongreen were cultured as described above and harvested by centrifugation at 3,000 *g* for 10 min. The cell pellet (approximately 100 µL) was resuspended in 2 mL of 2.5% (w/v) glutaraldehyde in CAB and fixed for 2 hr at RT with gentle rotating (20 rpm). Cells were pelleted by centrifugation at 6,000 *g* for 2 min and were washed twice for 10 min with 100 mM CAB. Cells were postfixed with 1% (w/v) osmium tetroxide in 100 mM CAB for 2 hr and subsequently washed twice with ddH2O. Cells were dehydrated by incubation in an ethanol gradient, 50% EtOH for 10 min, 70% EtOH overnight, and 90% EtOH for 10 min followed by three 10-min washes in 100% dry EtOH. Cells were then washed twice with propylene oxide for 15 min. Cell pellets were embedded by re- suspension in 1 mL of a 1:1 mix of propylene oxide and Agar LV Resin and incubated for 30 min with rotation. Cell pellets were infiltrated twice in 100% Agar LV resin (2 × 2h). The cell pellet was resuspended in fresh resin and transferred to a 1-mL BEEM embedding capsule, centrifuged for 5 min at 1,100 rpm in a swing out rotor to concentrate the cells in the tip of the capsule and samples were polymerised for 20 hr at 60°C.

Ultrathin sections were cut using a Leica EM UC7 ultramicrotome equipped with a diamond knife (DiATOME 45°). Sections (70 nm) were collected on uncoated 400-mesh copper grids. Grids were stained by incubation in 4.5% (w/v) uranyl acetate in 1% (v/v) acetic acid for 45 min followed by washing in a stream of ddH2O. Grids were then stained with Reynolds lead citrate for 7 min followed by washing in a stream of ddH2O. Electron microscopy was performed using a JEOL-1230 transmission electron microscope operated at an accelerating voltage of 80 kV equipped with a Gatan One View digital camera.

Immuno-EM of isolated vesicles

2 µL of filtered media containing recombinant vesicles from a culture of *E. coli* expressing VNp-mNeongreen was placed onto a formvar/carbon coated 600 mesh copper grid and left for 5 mins at room temperature to allow vesicles to settle. Vesicles were osmotically shocked to rupture vesicles by moving grids into 2 × 20 µL drops of milliQ water for 10 minutes at RT. Samples were then fixed in 2% formaldehyde and 0.5% glutaraldehyde in CAB for 15 minutes at RT. Grids were then washed in 6 × 20 µL drops of CAB and 6 × 20 µL drops of TBST (20 mM Tris-HCl, 500 mM NaCl, 0.05% Tween 20 and 0.1% BSA pH7.4). Samples were blocked in a 20 µL drop of 2% BSA in TBST at room temperature for 30 min. Grids were then transferred directly into a 20 µL drop of anti-mNeongreen rabbit polyclonal (Cell Signalling Technology) primary antibody diluted 1:100 in TBST and incubated for 1 hr. Grids were washed in

6 × 20 μL drops of TBST. Grids were then moved into a drop of goat anti-rabbit IgG 5 nm gold (British Biocell International) diluted 1:50 and then moved to a fresh drop of the same antibody and incubated for 30 min. Excess antibody was removed by washing in 6 × 20 μL drops of TBST and 6 × 20 μL drops of milliQ water and dried.

Grids were negative stained for 5 seconds in 2% aqueous uranyl acetate. Electron microscopy was performed using a JEOL-1230 transmission electron microscope operated at an accelerating voltage of 80 kV equipped with a Gatan One View digital camera.

Immuno-EM of *E. coli* cells

E. coli expressing VNp-mNeongreen were cultured as described above and harvested by centrifugation at 3,000 *g* for 10 min. The cell pellet (approximately 100 μL) was resuspended in 2 mL 2% (w/v) formaldehyde and 0.5% glutaraldehyde in CAB and fixed for 2h at RT. The sample was washed 2 ×10 minutes in CAB. Cells were dehydrated by incubation in an ethanol gradient, 50% EtOH for 10 min, 70% EtOH overnight, and 90% EtOH for 10 min followed by three 10-min washes in 100% dry EtOH. Cells were then suspended in LR White resin medium grade (London Resin Company) for 4h and then in fresh LR White resin overnight. Following 2 × 4h changes in fresh LR White resin samples were placed in sealed gelatine capsules and spun in a swing out rotor at 1,100 rpm to concentrate cells. Gelatine capsules containing the cell pellets were polymerised upright at 60°C for 20 hours. Ultrathin sections were cut using a Leica EM UC7 ultramicrotome equipped with a diamond knife (DiATOME 45°). Sections (80 nm) were collected on uncoated 400-mesh gold grids.

Samples were blocked in a 20 μL drop of 2% BSA in TBST at room temperature for 30 min. Grids were then transferred directly into a 20 μL drop of anti-mNeongreen rabbit polyclonal (Cell Signalling Technology) primary antibody diluted 1:10 in TBST and incubated for 1 hr. Grids were washed in 6 x TBST. Grids were then moved into a drop of goat anti-rabbit IgG 5 nm gold (British Biocell International) diluted 1:50 and then moved to a fresh drop of the same antibody and incubated for 30 min. Excess antibody was removed by washing in 6 × 20 μL drops of TBST and 6 × 20 μL drops of milliQ water and dried.

Grids were stained for 15 min in 4.5% uranyl acetate in 1% acetic acid solution and then washed in 6 × 20 μL drops of milliQ water. Grids were then stained with Reynolds lead citrate for 3 min and washed in 6 × 20 μL drops of milliQ water. Electron microscopy was performed using a JEOL-1230 transmission electron microscope operated at an accelerating voltage of 80 kV equipped with a Gatan One View digital camera.

QUANTIFICATION AND STATISTICAL ANALYSIS

Statistical details of experiments can be found in the figure legends, including a definition of exact values of n, and details of error bars.

Cell Reports Methods, Volume *3*

Supplemental information

High-yield vesicle-packaged recombinant

protein production from *E. coli*

Tara A. Eastwood, Karen Baker, Bree R. Streather, Nyasha Allen, Lin Wang, Stanley W. Botchway, Ian R. Brown, Jennifer R. Hiscock, Christopher Lennon, and Daniel P. Mulvihill

Supplemental Items

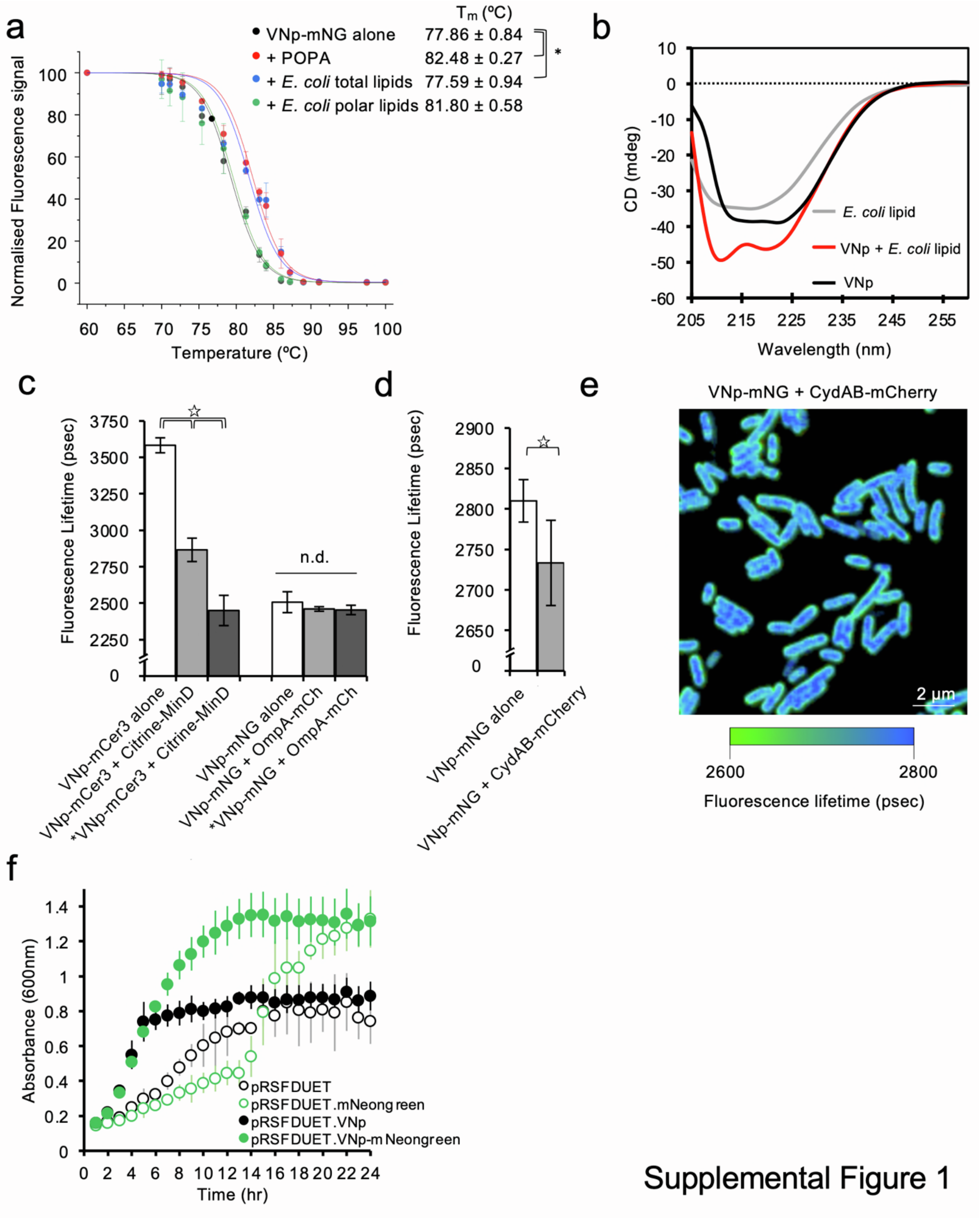

Supplemental Figure 1

SUPPLEMENTAL FIGURE 1. VNp interacts with the inner *E. coli* membrane. Related to Figure 1 and STAR Methods. Thermal shift (a) and Circular Dichroism (b) assays were used to confirm interaction between the VNp and membrane composed of *E. coli* membrane lipids *in vitro.* (a) Interaction with the membrane increases thermal stability of a membrane associated fluorophore. The thermal shift assay was used to examine the impact of different lipid membranes upon fluorophore signal from a fluorescent protein (mNeongreen) when fused to the VNp, a potential membrane binding protein. Average Thermal shift mNeongreen fluorescence curves were calculated for VNp-mNeongreen alone (black), and VNp-mNeongreen in the presence of 100 nm vesicles composed of phosphatidic acid (red), or mixtures of either total (blue) or polar (green) *E.coli* lipids. The shift to the right signifies the VNp-mNeongreen interacts with membranes composed of phosphatidic acid or a mixture of total *E. coli* lipids. (b) Circular Dichroism was used to examine the impact *E. coli* membrane binding has upon the predicted alpha-helical VNp structure. The graph shows averaged CD spectra of VNp alone (black), total *E. coli* lipid vesicles alone (grey), or from a mixture of VNp and *E. coli* lipid vesicles (red). The relative broad negative CD spectra peaks at 208 nm and 222 nm, observed in the mixture of VNp and *E. coli* lipid membrane are consistent with single α-helical structures, and these spectra show that the VNp alpha-helix is stabilised upon interaction with *E. coli* membrane lipid vesicles. (c) Single-photon and (d-e) Multi-photon Fluorescence Lifetime Imaging based Fluorescence Resonance Energy Transfer Microscopy (FLIM-FRET) was used to examine physical interactions between VNp and the *E. coli* inner and outer membranes *in vivo*. Cerulean3 (VNp-Cer3) or mNeongreen (VNp-mNG) fluoreophores were used as donors, and Citrine (Citrine-MinD) or mCherry (OmpA-mCherry/CydAB-mCherry) were used as acceptors. FRET dependent reduction in the fluorescence lifetime of the donor indicates physical interaction (< 10 nm) between proteins (☆ - 99.99% confidence levels). (c) Histogram of donor fluorophore Fluorescence lifetimes of *E. coli* cells expressing VNp donor fluorophore fusions (VNp-Cer3 / VNp-mNG) either alone or with acceptor fluorophore labelled inner (Citrine-MinD) or outer (OmpA-mCherry) membrane proteins indicate VNp interacts with the *E. coli* inner membrane. (d) The histogram of mNeongreen fluorescence lifetime within *E. coli* cells expressing an mNeongreen donor fluorophore VNp fusion (VNp-mNG alone or in combination with the mCherry labelled CydAB inner membrane complex confirmed interaction between VNp and the inner membrane. (e) mNeongreen fluorescence Lifetime micrograph of VNp-mNG CydAB-mCherry expressing cells (from d) illustrates the reduced fluorescence lifetime of the VNp-mNeongreen at the cell membrane, where CydAB is located. The reduction in lifetime length reflected in change from blue (2.8 ns) to green (2.6 ns). (f) To assess the impact of VNp expression on *E. coli* viability averaged growth curves were generated from 4 independent replicate cultures of BL21(DE3) *E. coli* cells containing either an empty pRSFDUET vector (empty black circles), pRSFDUET.mNeongreen (empty green circles), pRSFDUET.VNp (filled black circles) or pRSFDUET.VNp.mNeongreen (filled green circles). Cells were grown at 37 ºC in TB supplemented with kanamycin and 20 μg/ml IPTG on the same 96-well plate. Vesicle containing centrifuged media had no measurable difference in absorbance at 600 nm, indicating the observed changes in absorbance are due to increase in cell number. These data illustrate expression of VNp or a VNp fusion does not negatively impact bacterial growth over a 24 hour period.

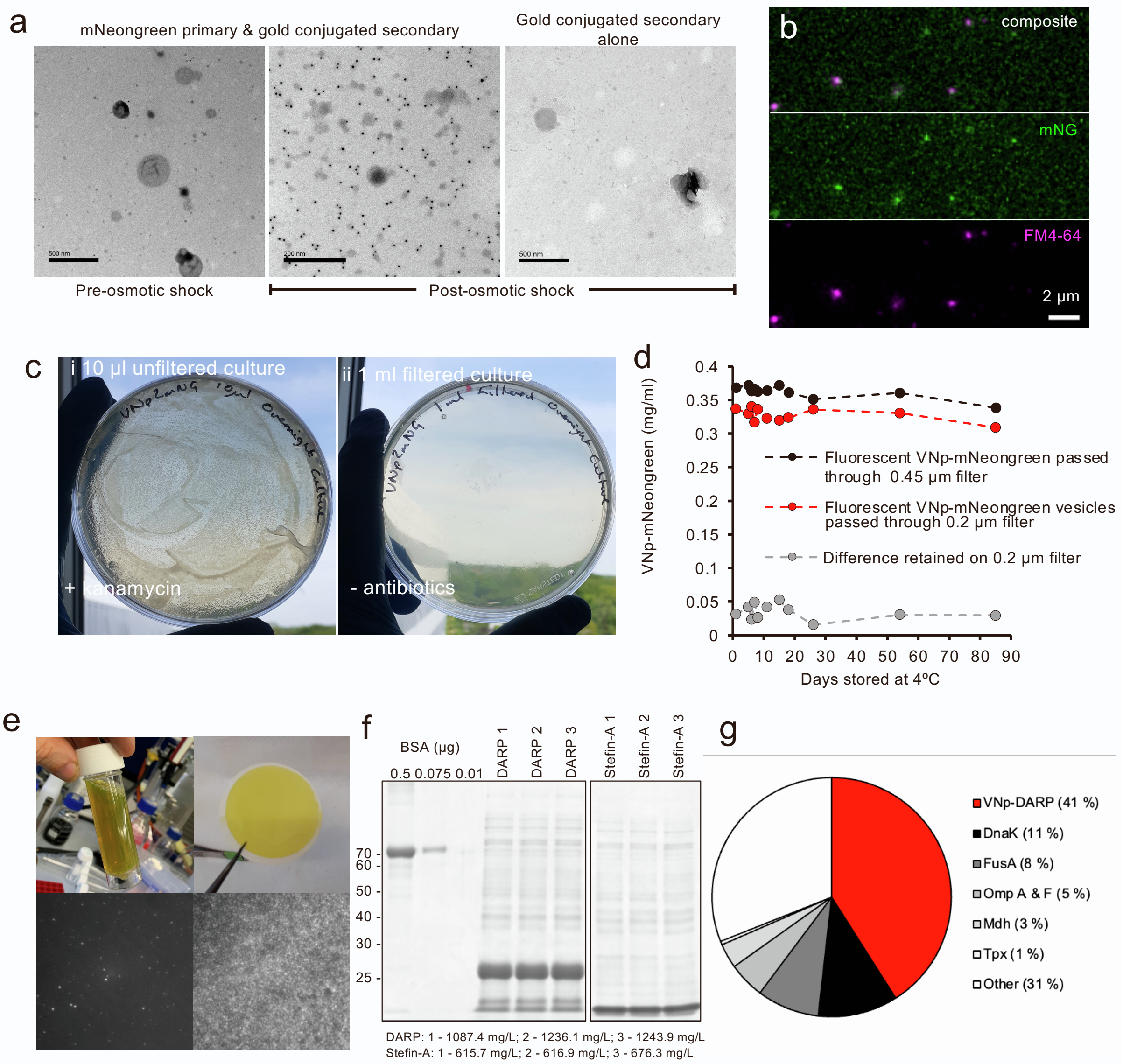

Supplemental Figure 2

Supplemental Figure 2: VNp fusion protein is contained within the lumen of isolated recombinant vesicles. Related to STAR Methods. VNp-mNeongreen containing vesicles were filter purified from media of an overnight culture of BL21 DE3 pRSFDUET1-VNp-mNeongreen cells and mounted onto EM grids and subjected to anti-mNeongreen immuno EM analysis. VNp-mNeongreen dependent gold labelled densities bound to mNeongreen released from vesicles upon osmotic shock from resuspension in water. The lack of densities in control samples subjected to either immuno-analysis prior to bursting, or burst vesicles processed in the same way but without primary anti-mNeongreen antibodies illustrate the mNeongreen is located exclusively within the lumen of the vesicles. (b) Wide field image of VNp-mNeongreen (green) vesicles subjected to FM4-64 (magenta) membrane staining. (c) Test illustrating exclusion of viable *E. coli* cells from the vesicle containing filtrate. 10 µl of total culture (i) and 1 ml of 0.45µm media filtrate (ii) from an overnight culture of VNp-mNG expressing *E. coli* cells were plated out onto LB (ii) or LB supplemented with kanamycin (i) and incubated overnight at 37 ºC. (d) The VNp induced vesicle provided a stable environment for storage of VNp-fusions. VNp-mNeongreen containing vesicles, filter purified from media of an overnight culture of BL21 DE3 pRSFDUET1-VNp-mNeongreen cells were stored at 4 ºC. Overall mNeongreen fluoresecence and the fraction of mNeongreen fluorescence within vesicles retained by a 0.2µm filter did not vary over time, indicating stability of vesicles and folded mNeongreen protein within them. (e) Universal containing centrifuged media from an overnight culture of VNp-mNeongreen expressing cells (upper left) concentrated upon 0.1 µm filter (top right), and associated wide-field flourescence images of mNeongreen containing vesicles. (f) SDS-PAGE gel of BSA quantitation controls and 8 µl of centrifuged media fractions from VNp-fusion induction cultures from independent biological repeats. These samples were subject to tryptic digest and proteomic analysis (example VNp-DARP1 sample1 shown in Table S1). (g) Pie chart showing typical composition of VNp-fusion containing vesicles as determined from the proteomic analysis .

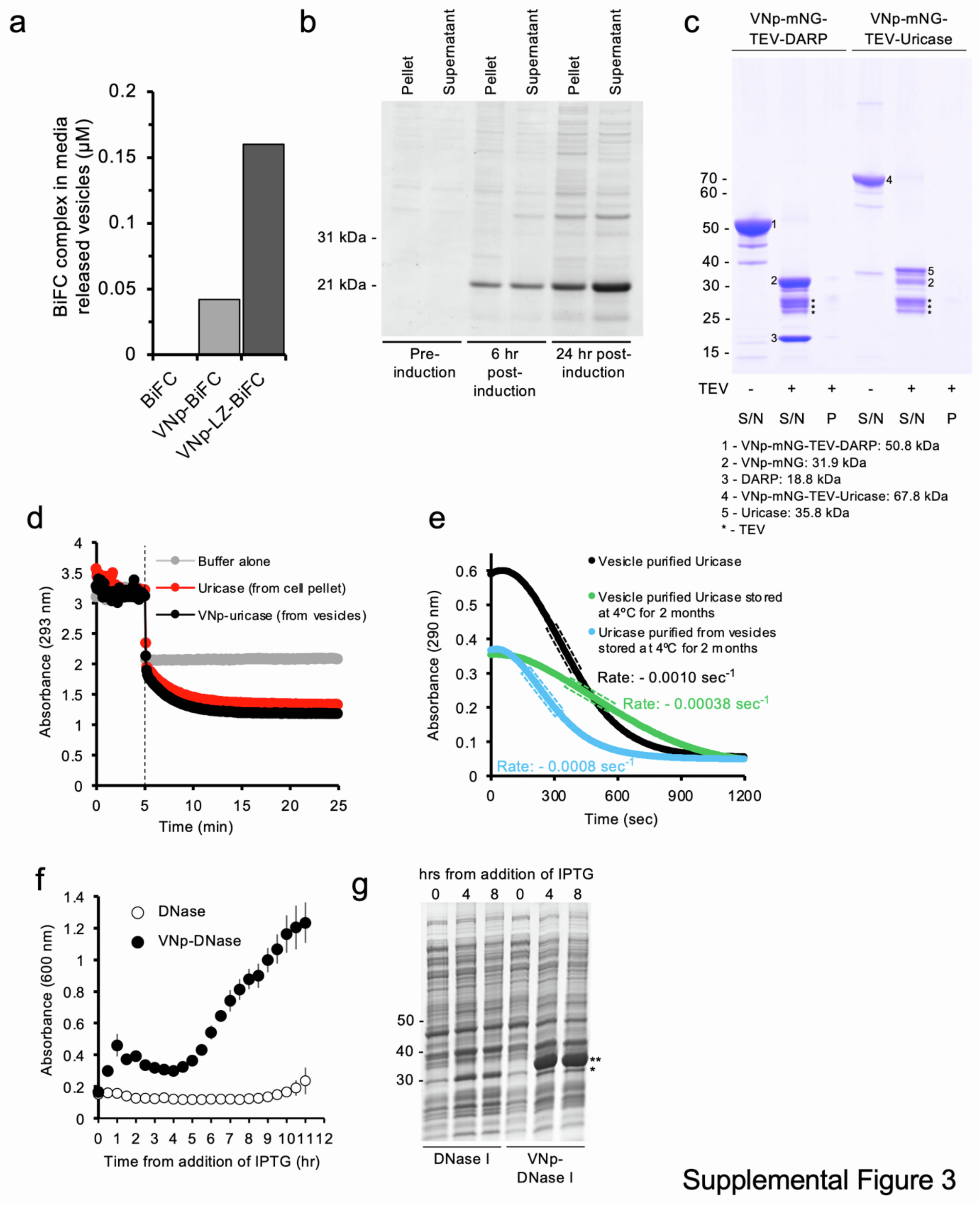

Supplemental Figure 3

SUPPLEMENTAL FIGURE 3: Functional VNp-fusions are targeted to the VNp-vesicles. Related to STAR Methods. (a) Bimolecular Fluorescence Complementation dependent Fluorescence between the amino (VenusN154) and carboxyl (VenusC155) was used to establish heterocomplexes between different VNp fusions could be targeted to and isolated from the same vesicle. VenusN154-VenusC155 BiFC dependent fluorescence (515 nm) from vesicles isolated from BL21(DE3) *E. coli* expressing either VenusN154 & VenusC155 (white); VNp-VenusN154 & VNp-VenusC155 (grey); or VNp-LZ-VenusN154_VNp-LZ-VenusC155 (black) was used to establish the concentration of the BiFC complex within isolated vesicles. (b) Coomassie stained SDS-PAGE gel of cell pellet and filtered media supernatant samples taken from a 24 hr 15 L fermenter culture of BL21-DE3 cells expressing VNp-DARP. Obtained VNp-DARP yields at end of fermentation were 1.4 g/L in media and 3.1 g/L in cell pellets. (c) Purified VNp-mNG-TEV-DARP and VNp-mNG-TEV-Uricase were digested with TEV protease. The resultant cleaved proteins were not detectable within the pellet fraction (P), and remained in the supernatant (S/N) fraction after centrifugation at 13,200 RCF. (d) The enzymatic activity of Uricase either isolated from a cell pellet using conventional methods (red) or from VNp-uricase induced vesicles (black) was examined to compare functionality of each protein. A buffer only control (grey) shows dilution dependent change in baseline 293 nm absorbance of uric acid. Uricase enzyme / buffer was added to the uric acid substrate after a 5 min equilibration (dashed line). The activity of uricase isolated from cell pellet or VNp-uricase containing vesicles were equivalent. (e) To examine the stability of uricase enzyme activity from protein stored within VNp-uricase vesicles, uricase activity of either fresh vesicle purified VNp-uricase (black; the same vesicle purified VNp-uricase stored in reaction buffer at 4 ºC for 2 months (green); or freshly purified from vesicles that had been stored at 4 ºC for 2 months (blue), were measured using stopped-flow. Rates were determined from steady state regions (highlighted by boxes) of averaged curves and show while uricase stored in buffer exhibited 38% of the original activity, uricase stored within vesicles retained 80% of the original enzymatic activity. (e & f) To establish whether vesicular compartmentalisation of VNp fusions allowed expression of toxic proteins the expression and impact on *E. coli* growth of DNAse1 and VNp-DNaseI were compared. (a) Average growth curves and (b) expression profiles of *E. coli* expressing DNase and VNp-DNase show that while DNaseI only had minimal expression, it inhibited growth of the *E. coli* cells. In contrast the VNp-DNAseI expressed cells grew normally and expressed meaningful levels of the fusion protein.Predicted sizes of DNase and VNp-DNase are 30.2 and 34.4 kDa respectively.

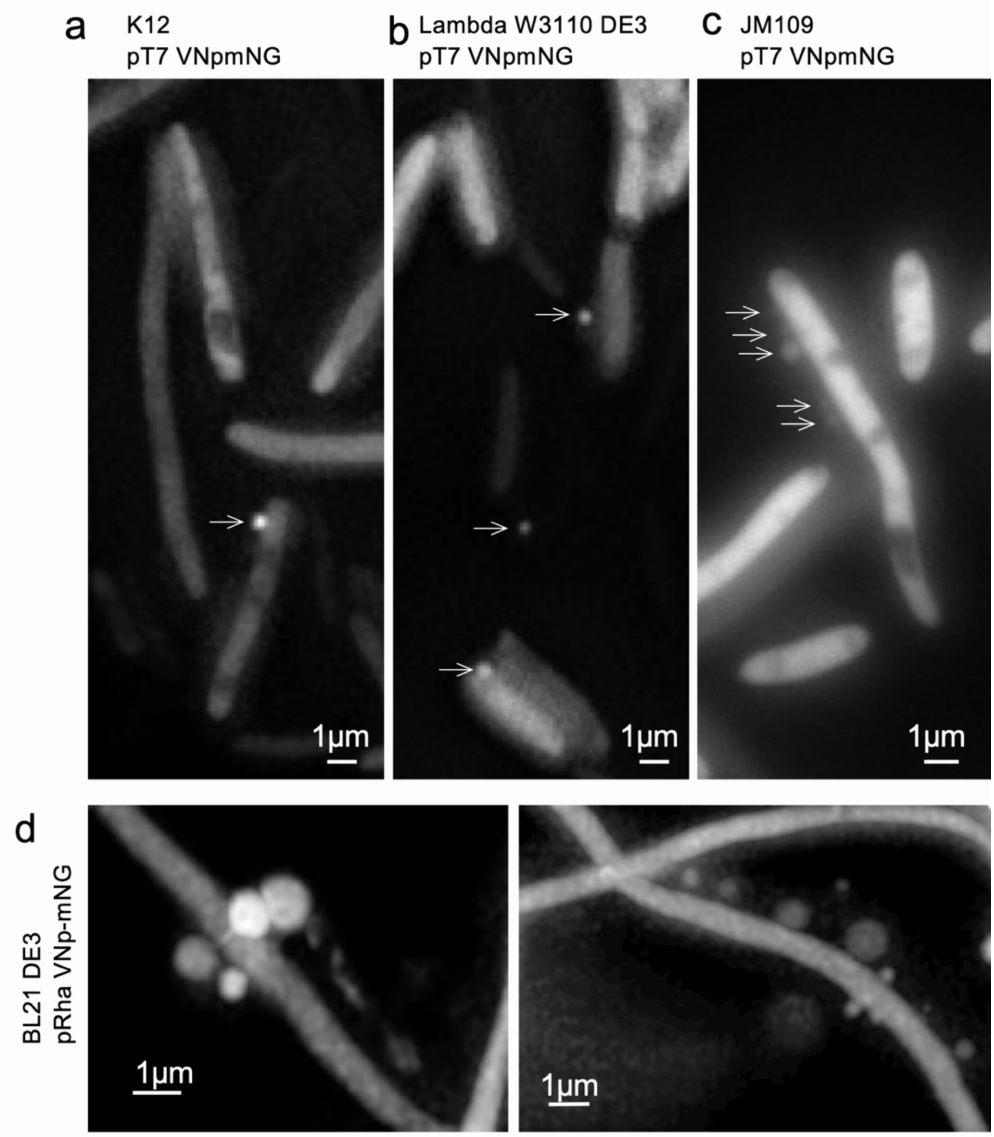

e *Yields of exported protein from W3110 lambda DE3 E. coli cells*

VNp-fusion	Yields (mg protein / L culture)
VNp-DARP	262.8 ± 35.1
VNp-mNeongreen	128.2
VNp6-mNeongreen	478.8
VNp15-mNeongreen	445.4

SUPPLEMENTAL FIGURE 4: VNp induced recombinant protein packaged vesicles using different *E. coli* strains and promoters. Relates to Table 1. Widefield fluorescence images of (a) K12, (b) Lambda DE3 W3110, (c) JM109, and (d) BL21 DE3 *E. coli* strains expressing VNp-mNeongreen from the T7 (a, b, c)) or Rhamnose (d) promoters. (e) Exported Yields of VNp-fusions from W3110 lambda cells.

Supplemental Table 1: Proteomic analysis of purified VNp-DARP induced vesicles. Proteomic data from analysis of purified VNp-DARP containing recombinant vesicles (Sample DARP1 from S5b). Vesicle sample was subjected to tryptic digest and mass-spectroscopic analysis. Related to STAR Methods and supplemental figure 2g.

Gene name/Protein	Unique Peptide count	Confidence score	Mass	Raw abundance	Relative abundance
VNp2-DARP	11	94.9	22503	2578835	41.00
dnaK / Chaperone protein DnaK	29	201.2	69172	679166	10.80
fusA / Elongation factor G	38	250.2	77753	530543	8.43
ompF & ompA / Outer membrane porin	12	80.8	39333	309297	4.92
mdh / Malate dehydrogenase	17	121.0	32508	209716	3.33
malE / Maltose/maltodextrin-binding periplasmic protein	10	67.5	43388	166822	2.65
tsf / Elongation factor Ts	13	88.1	30537	115992	1.84
gapA / Glyceraldehyde-3-phosphate dehydrogenase A	7	46.0	35704	109823	1.75
ahpC / Alkyl hydroperoxide reductase C	5	38.3	20876	98732	1.57
grpE / Protein GrpE	2	13.2	21798	65577	1.04
groES / Co-chaperonin GroES	2	12.7	10387	63804	1.01
fbaA / Fructose-bisphosphate aldolase class 2	4	24.4	39375	60187	0.96
eno / Enolase	11	66.9	45712	57412	0.91
slyD / FKBP-type peptidyl-prolyl cis-trans isomerase SlyD	3	20.7	21195	53276	0.85
pgk / Phosphoglycerate kinase	9	58.4	41289	52081	0.83
adk / Adenylate kinase	8	49.6	23643	51143	0.81
tufA / Elongation factor Tu 1	8	53.3	43455	49205	0.78
cysK / Cysteine synthase A	7	58.7	34547	40317	0.64
yncE / Uncharacterized protein YncE	2	10.3	38613	39498	0.63
glnH / Glutamine-binding periplasmic protein	2	11.3	27190	39391	0.63
fabI / Enoyl-[acyl-carrier-protein] reductase [NADH] FabI	5	26.6	28092	39285	0.62
rplL / 50S ribosomal protein L7/L12	1	5.8	12295	38687	0.62
sodB / Superoxide dismutase [Fe]	2	11.2	21323	37129	0.59
frr / Ribosome-recycling factor	1	6.4	20696	31271	0.50
tpx / Thiol peroxidase	3	19.9	18006	30978	0.49
tnaA / Tryptophanase	7	44.0	53173	30304	0.48

lamB / Maltoporin	7	40.1	50026	27768	0.44
cysP / Thiosulfate-binding protein	5	30.2	37615	24683	0.39
glpQ / Glycerophosphodiester phosphodiesterase	4	21.9	40900	24594	0.39
serS / Serine-tRNA ligase	3	17.1	48699	23970	0.38
gpmA / 2_3-bisphosphoglycerate-dependent phosphoglycerate mutase	2	12.6	28556	23620	0.38
glyA / Serine hydroxymethyltransferase	3	16.7	45488	23175	0.37
pal / Peptidoglycan-associated lipoprotein	1	6.3	18881	23114	0.37
aspC / Aspartate aminotransferase	6	35.9	43859	23029	0.37
oppA / Periplasmic oligopeptide-binding protein	8	47.2	61013	22230	0.35
mlaC / Intermembrane phospholipid transport system binding protein MlaC	4	25.2	23963	21725	0.35
crr / PTS system glucose-specific EIIA component	5	29.1	18251	18180	0.29
ahpF / Alkyl hydroperoxide reductase subunit F	2	10.8	56519	17356	0.28
tolB / Tol-Pal system protein TolB	4	25.3	45956	17290	0.27
trxA / Thioredoxin 1	2	11.2	11921	17188	0.27
hisJ / Histidine-binding periplasmic protein	2	12.2	28597	16840	0.27
fkpA / FKBP-type peptidyl-prolyl cis-trans isomerase FkpA	5	28.0	28882	16840	0.27
talB / Transaldolase B	4	24.0	35390	16519	0.26
htpG / Chaperone protein HtpG	5	28.0	71423	16315	0.26
uspA / Universal stress protein A	1	6.7	16123	16070	0.26
gcvP / Glycine dehydrogenase (decarboxylating)	4	21.4	105118	16057	0.26
ridA / 2-iminobutanoate/2-iminopropanoate deaminase	1	6.1	13669	15454	0.25
groEL / Chaperonin GroEL	4	23.0	57500	14988	0.24
potD / Spermidine/putrescine-binding periplasmic protein	2	11.3	38867	14922	0.24
sspA / Stringent starvation protein A	3	17.8	24362	14780	0.23
ackA / Acetate kinase	1	5.4	43633	13679	0.22
dapD / 2_3_4_5-tetrahydropyridine-2_6-dicarboxylate N-succinyltransferase	4	24.0	30063	13347	0.21
glpK / Glycerol kinase	2	11.6	56516	12207	0.19
rbsB / Ribose import binding protein RbsB	2	10.4	30951	11419	0.18

yiaD / Probable lipoprotein YiaD	2	10.5	22254	11288	0.18
pfkB / ATP-dependent 6-phosphofructokinase isozyme 2	1	5.3	32684	11133	0.18
agp / Glucose-1-phosphatase	4	21.3	46025	10709	0.17
asnS / Asparagine-tRNA ligase	5	26.4	52799	9952	0.16
tktA / Transketolase 1	3	17.1	72497	9776	0.16
valS / Valine-tRNA ligase	4	21.3	108649	9747	0.15
lysU / Lysine-tRNA ligase_ heat inducible	4	20.9	57884	9668	0.15
accB / Biotin carboxyl carrier protein of acetyl-CoA carboxylase	1	5.0	16744	8273	0.13
lpp / Major outer membrane lipoprotein Lpp	2	11.7	8381	7809	0.12
yajQ / UPF0234 protein YajQ	1	6.0	18344	7291	0.12
fabA / 3-hydroxydecanoyl-[acyl-carrier-protein] dehydratase	1	5.4	19083	7240	0.12
sucC / Succinate-CoA ligase [ADP-forming] subunit beta	3	16.8	41678	7220	0.11
trxB / Thioredoxin reductase	3	18.4	34851	7083	0.11
hemC / Porphobilinogen deaminase	1	5.8	34080	7060	0.11
degP / Periplasmic serine endoprotease DegP	3	17.0	49468	6886	0.11
yajG / Uncharacterized lipoprotein YajG	1	5.7	21007	6703	0.11
rpsA / 30S ribosomal protein S1	5	27.4	61272	6484	0.10
pykF / Pyruvate kinase I	1	5.3	51072	5739	0.09
moaD / Molybdopterin synthase sulfur carrier subunit	1	5.5	8758	5737	0.09
borD / Prophage lipoprotein Bor homolog	1	5.6	10618	5555	0.09
upp / Uracil phosphoribosyltransferase	1	4.9	22590	5081	0.08
yraP / Uncharacterized protein YraP	1	5.8	20085	5055	0.08
nfuA / Fe/S biogenesis protein NfuA	1	5.5	21226	4614	0.07
pta / Phosphate acetyltransferase	4	20.7	77514	4481	0.07
gloC / Hydroxyacylglutathione hydrolase GloC	1	5.7	24012	4267	0.07
yfbU / UPF0304 protein YfbU	2	10.6	19650	3166	0.05
nusA / Transcription termination/antitermination protein NusA	1	4.9	55042	2767	0.04
tig / Trigger factor	1	5.5	48193	1989	0.03
ppiB / Peptidyl-prolyl cis-trans isomerase B	1	6.2	18268	1654	0.03

Supplemental Video 1: SIM time-lapse of BL21 DE3 containing induced pRSFDuet-1_VNp-LZ_CydAB-mNeongreen. CydAB-mNeongreen labelled inner membranes highlight dynamic movement of VNp-LZ fusion induced membrane bound cytosolic vesicles (100 msec / frame).
